# Divisive normalization processors in the early visual system of the *Drosophila* brain

**DOI:** 10.1007/s00422-023-00972-x

**Published:** 2023-09-13

**Authors:** Aurel A. Lazar, Yiyin Zhou

**Affiliations:** 1 Department of Electrical Engineering, Columbia University, New York, NY 10027 USA; 2 Present Address: Department of Computer and Information Science, Fordham University, New York, NY 10023 USA

**Keywords:** Motion detection, Divisive normalization, *Drosophila*, Phase processing, Gain control

## Abstract

Divisive normalization is a model of canonical computation of brain circuits. We demonstrate that two cascaded divisive normalization processors (DNPs), carrying out intensity/contrast gain control and elementary motion detection, respectively, can model the robust motion detection realized by the early visual system of the fruit fly. We first introduce a model of elementary motion detection and rewrite its underlying phase-based motion detection algorithm as a feedforward divisive normalization processor. We then cascade the DNP modeling the photoreceptor/amacrine cell layer with the motion detection DNP. We extensively evaluate the DNP for motion detection in dynamic environments where light intensity varies by orders of magnitude. The results are compared to other bio-inspired motion detectors as well as state-of-the-art optic flow algorithms under natural conditions. Our results demonstrate the potential of DNPs as canonical building blocks modeling the analog processing of early visual systems. The model highlights analog processing for accurately detecting visual motion, in both vertebrates and invertebrates. The results presented here shed new light on employing DNP-based algorithms in computer vision.

**Supplementary Information:**

The online version contains supplementary material available at 10.1007/s00422-023-00972-x.

## Introduction

Robust motion detection is the key first processing step for insects to safely navigate complex environments. Current state-of-the-art computer vision algorithms achieve good performance under demanding navigation conditions. However, under extreme conditions, their performance often quickly degrades (Mathis et al. 2016; Li et al. 2018). Surprisingly, however, the early vision system of the fruit fly is remarkably accurate at detecting motion in complex environments under a vast range of light intensity conditions. The logic of computation in the fly visual system is substantially different from the traditional methods of computation employed by current man-made counterparts. This enables the fly to navigate through terrains with rapid light intensity changes, even though it only possesses a single photoreceptor type (van Hateren 1997).

**Fig. 1:**
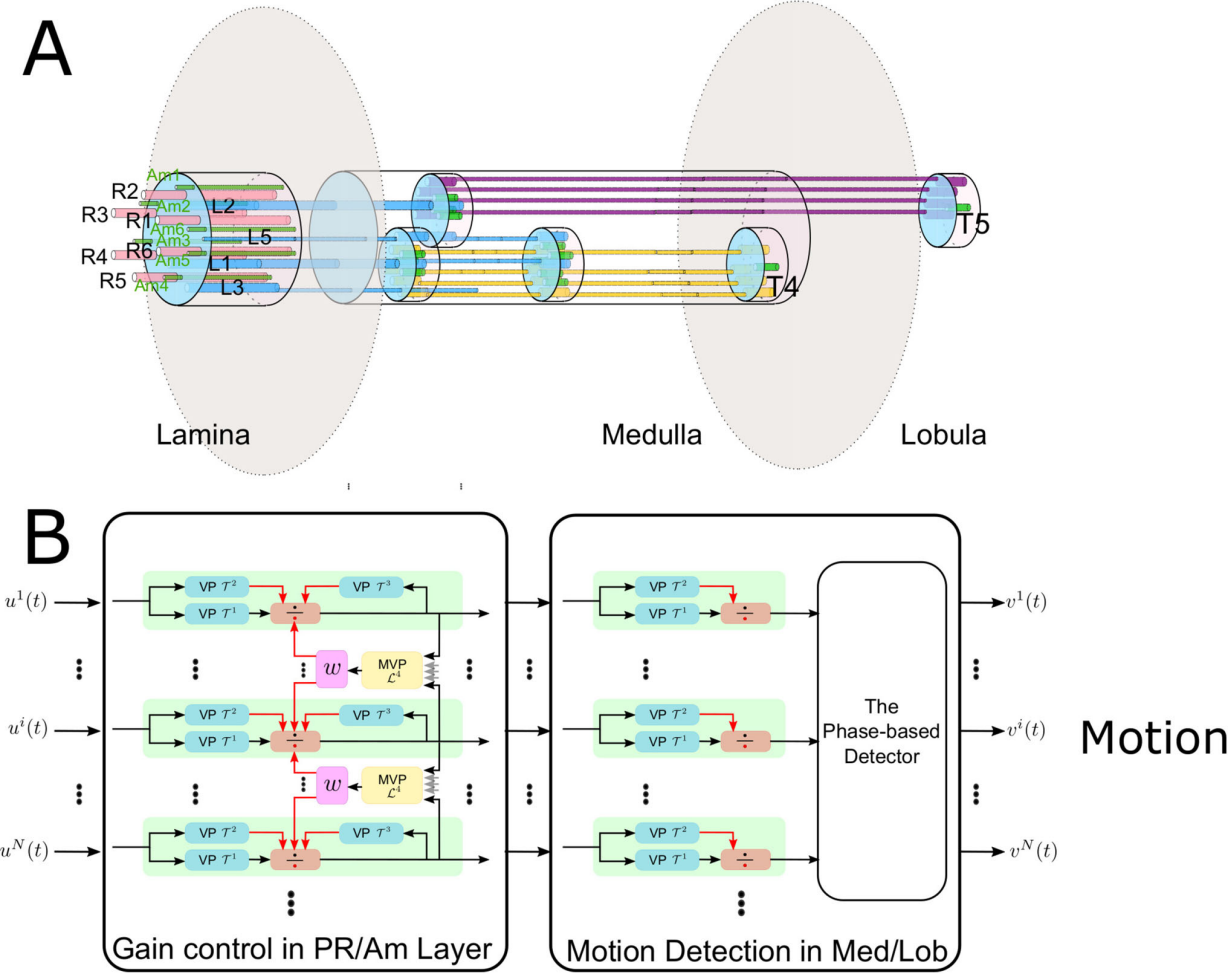
End-to-end motion detection pathways in the early vision system of the fruit fly and a cascade of two DNPs modeling the motion detection circuit. **A** Canonical neural circuits with components embedded into the motion detection pathway. Canonical circuits in each neuropil are indicated by wide, long cylinders. Flat cylinders represent the intersection of canonical circuits with processing layers such as strata in the Medulla and layers in the Lobula. Thin, long cylinders represent the neurites of (pink) photoreceptors, (blue) Lamina output neurons, (yellow) ON-pathway neurons, (violet) OFF-pathway neurons, (green) neurites of wide-field neurons that innervate multiple canonical circuits in the same stratum/layer. **B** A cascade of two DNP blocks modeling the motion detection pathways in the fly eye. The first DNP block (left) models gain control of visual stimuli in the photoreceptor/amacrine cell layer, corresponding to the left cylinder in (**A**). The second block (right) models the motion detection in the Medulla/Lobula, corresponding to the right cylinder in (**A**)

Modern optic flow algorithms may take seconds or even minutes to compare two consecutive frames (Baker et al. 2011; Menze et al. 2018). While the processing speed was recently improved upon by using deep neural network-based algorithms, the cost of training time and the required large amounts of training data remain excessive. For the low level tasks such as elementary motion detection, fly vision is far more efficient, faster and more robust without loss of precision during events that are critical for survival, such as rapid predator attacks taking place on short time scales (hundreds of milliseconds). Strikingly, in fruit flies, like in many other insects and mammals, processing delays are minimal. It only takes 3 synapses from photoreceptors to reach the neurons responsible for detecting low-level directional motion with minimal energy expenditure (Sy et al. 2013) ${yyz:}$(see Fig. 1A); an efficient computational principle of motion detection seems to be at work.

This calls for developing biologically informed robust motion detection algorithms. Two half-century-old computational theories of motion detection, namely the Reichardt motion detector (Hassenstein and Reichardt 1956) and Barlow–Levick motion detector (Barlow and Levick 1965), have dominated the field. Recent studies have unveiled the basic anatomical structure of the fly’s motion detection pathways (Yang and Clandinin 2018; Borst et al. 2020). While these and other studies did rapidly advance our understanding of motion detection in the early vision system of the fruit fly, the underlying models have yet to be successful in capturing the surprising robustness of fly vision. In Lazar et al. (2016), we compared the two prevailing models of fly motion detection with a more complex phase-based algorithm that we devised. Under different luminance and contrast conditions, we demonstrated that (i) none of the three algorithms could fully account for motion in natural scenes, and (ii) the detection of motion was not robust at low luminance/contrast levels. This suggests fundamental limits in current modeling approaches to visual motion detection that are narrowly focused on simple feedforward motion detection mechanisms. The latter do not match the vastly superior performance of the motion detection circuits in flies.

There is strong evidence showing that divisive normalization may contribute to gain control in olfaction (Olsen et al. 2010), vertebrate retina (Beaudoin et al. 2007), primary visual cortex (Carandini and Heeger 1994), primary auditory cortex (Rabinowitz et al. 2011) and sensory integration (Ohshiro et al. 2017). Feedforward divisive normalization has been proposed as a model of canonical neural computation (Carandini and Heeger 2012), and used in nonlinear image representation for achieving perceptual advantages (Lyu and Simoncelli 2008). This computation is key to many sensory processing circuits underlying adaptation and attention. Feedforward normalization is also frequently used in deep neural networks (Goodfellow et al. 2016; Ioffe and Szegedy 2015).

In Lazar et al. (2020), we presented a class of divisive normalization processors (DNPs) that operate in the time and the space-time domain. A DNP example is shown in the left block of Fig. 1B. Each DNP channel exhibits Volterra processors (VP) in a feedforward and local feedback divisive normalization branch. In addition, a multi-input Volterra processor (MVP) provides global feedback. With input from all channel outputs, the MVP provides feedback into each channel. This type of MIMO circuit architecture has been observed in many neural systems (Lazar et al. 2022b).

Stimuli processed by DNPs can be faithfully recovered from the output (Lazar et al. 2022a), suggesting that no information is lost during processing. We posit that a feedforward/feedback divisive normalization processor is embedded in every layer of the motion detection pathway (see Fig. 1A), including the lamina, medulla and a single layer in the lobula.

In this paper, we demonstrate that two key processing steps in the motion detection pathway, including the elementary motion detector and the intensity and contrast gain control mechanism, can be effectively modeled with DNPs. Two cascaded DNPs depicted in Fig. 1B implementing intensity and contrast gain control and elementary motion detection, respectively, can model the robust motion detection realized by the early visual system of the fruit fly brain. This suggests that, despite its nonlinearity, the class of DNPs can be used as computational building blocks in early sensory processing (Lazar et al. 2023).

This paper is organized as follows. In Sect. 2, we model the local phase-based motion detector devised in Lazar et al. (2016) using DNPs, and present an improved method for extracting motion velocity and derive an accuracy condition. In Sect. 3, we characterize the I/O of the DNP modeling the intensity and contrast gain control of the photoreceptor/amacrine cell layer, and evaluate its performance. In Sect. 4, we extensively evaluate the DNP for motion detection and the end-to-end motion detection performance with intensity and contrast gain control in natural, dynamic environments.

## Modeling motion detection with divisive normalization processors

In this section, we model the local phase-based motion detector (Lazar et al. 2016) as a DNP. This formulation provides a new insight into the structure of the local phase-based motion detector, and how phase computation can be carried out in neural circuits. With the DNP model of motion detection, we can directly relate the phase-based motion detector to existing bio-inspired motion detection models such as the Reichardt motion detector (Hassenstein and Reichardt 1956) and the motion energy model (Adelson and Bergen 1985). We then provide a new way for extracting both direction and velocity information as well as a new estimate for motion, and discuss the condition for the estimate to be accurate. Finally, we illustrate the motion detector’s sensitivity to brightness and contrast levels and demonstrate its limitations.

### Divisive normalization underlying the local phase-based motion detector

Let $(x_0, y_0)$ be an arbitrary point of reference of the visual field. The Fourier transform of the stimulus *u*(*x*, *y*, *t*) projected onto the Gaussian window function centered at $(x_0,y_0)$ and denoted by $w_{x_0, y_0}(x,y) = e^{-\left( (x-x_0)^2+(y-y_0)^2\right) /2\sigma ^2}$ is given by

1 $$\begin{aligned} \begin{aligned} U_{x_0, y_0}(\omega _x, \omega _y, t)&= \int _{{\mathbb {R}}^2} u(x,y,t) w_{x_0, y_0}(x,y) \\&\quad e^{-j(\omega _x (x-x_0) + \omega _y (y-y_0))}\text {d}x \text {d}y \\&= a_{x_0,y_0}(\omega _x, \omega _y, t) + j b_{x_0,y_0}(\omega _x, \omega _y, t) \\&= A_{x_0, y_0}(\omega _x, \omega _y, t) e^{j\phi _{x_0, y_0} (\omega _x, \omega _y, t)}, \end{aligned}\nonumber \\ \end{aligned}$$

where $a_{x_0,y_0}(\omega _x, \omega _y, t)$ and $b_{x_0,y_0}(\omega _x, \omega _y, t)$, respectively, are outputs of a quadrature pair of Gabor receptive fields with input stimulus *u*(*x*, *y*, *t*). Furthermore, $A_{x_0, y_0}(\omega _x, \omega _y, t)$ and $\phi _{x_0, y_0} (\omega _x, \omega _y, t)$ are, respectively, the *local amplitude* and *local phase* defined at location $(x_0, y_0)$ as a function of the frequency pair $(\omega _x, \omega _y)$ and time (*t*).

The local phase of the stimulus at position $(x_0, y_0)$ and frequency $(\omega _x, \omega _y)$ defined in Eq. (1), can be computed as (see the Appendix A.1 for the definition of ${\text {arctan2}}$)

2 $$\begin{aligned}{} & {} \phi _{x_0,y_0}(\omega _x, \omega _y, t) \nonumber \\{} & {} \quad = {\text {arctan2}}\left( b_{x_0, y_0}(\omega _x, \omega _y, t), a_{x_0, y_0}(\omega _x, \omega _y, t)\right) , \end{aligned}$$

The time derivative of the local phase $\phi _{x_0,y_0}(\omega _x, \omega _y,t)$ amounts to

3 $$\begin{aligned} \frac{\text {d}\phi _{x_0, y_0}}{\text {d}t}(\omega _x, \omega _y, t) = \frac{{\mathcal {T}}^1u (x,y,t)}{{\mathcal {T}}^2u (x,y,t)} , \end{aligned}$$

where

4 $$\begin{aligned} {\mathcal {T}}^1u (x,y,t)= & {} a_{x_0,y_0}(\omega _x, \omega _y, t) \frac{\text {d}b_{x_0,y_0}}{\text {d}t} \nonumber \\{} & {} - b_{x_0,y_0}(\omega _x, \omega _y, t) \frac{\text {d}a_{x_0,y_0}}{\text {d}t} \end{aligned}$$

and

5 $$\begin{aligned} {\mathcal {T}}^2u (x,y,t) = a^2_{x_0,y_0}(\omega _x, \omega _y, t) + b^2_{x_0,y_0}(\omega _x, \omega _y, t) + \varepsilon .\nonumber \\ \end{aligned}$$

Here $\varepsilon$ is a small constant added to avoid division by zero. In other words, the change of phase at location $(x_0, y_0)$ as a function of the frequency $(\omega _x, \omega _y)$ is a feedforward DNP. Thus, the phase-based motion detector circuit briefly reviewed in Appendix A.2 is a Divisive Normalization Processor. Note also that in Appendix A.2, the time derivative of the local phase $\phi _{x_0,y_0}(\omega _x, \omega _y,t)$ is directly computed in (36).

We also note the striking similarity between the functional form of ${\mathcal {T}}_1$ and the *elaborated* Reichardt detector, briefly reviewed in Appendix A.1, long considered to be a model of elementary motion detection in insects (Hassenstein and Reichardt 1956). The numerator also takes the exact form of opponent energy in the motion energy model (Adelson and Bergen 1985) when the temporal filter is properly chosen. Note that the motion energy model is equivalent to the elaborated Reichardt detector (van Santen and Sperling 1985). The former has been widely used as a model of motion detector in the mammalian brain (Grzywacz et al. 1990; Simoncelli and Heeger 1998; Burge and Geisler 2015).

Due to the nature of second-order Volterra processing of the Reichardt and the motion energy detectors, their responses strongly depend on the brightness and contrast of the input visual scene. While the motion energy detector extracts the amplitude of a visual scene for certain *spatio-temporal* frequencies, the divisive normalization processor extracts the gradient of phase at certain *spatial* frequencies that are independent of the amplitude. Note that processing of the latter is still spatio-temporal.

### Estimating the magnitude of velocity and the direction of motion

In Lazar et al. (2016), we devised a criterion to detect motion and the direction of motion based on the Phase Motion Indicator (PMI), a construct built upon the Radon transform of the derivative of phase across all frequencies. We extend the construct here to extract both the direction and velocity of motion, and derive the condition when velocity can be estimated accurately.

We first remind the reader about the method of computation of the Radon transform of the derivative of the phase. We evaluate the Radon transform of $\frac{\text {d}\phi _{x_0,y_0}}{\text {d}t}(\omega _x, \omega _y, t)$ over a circular bounded domain $C = \left\{ (\omega _x, \omega _y) \vert \omega ^2_x+\omega ^2_y \le r^2 \right\}$ as

6 $$\begin{aligned} \begin{aligned}&\left( {\mathcal {R}} \frac{\text {d}\phi _{x_0,y_0}}{\text {d}t}\right) (\rho , \theta , t) = \int _{{\mathbb {R}}} \frac{\text {d}\phi _{x_0,y_0}}{\text {d}t} \left( \rho {\text {cos}}\theta + s {\text {sin}}\theta , \rho {\text {sin}}\theta \right. \\&\quad \left. - s {\text {cos}}\theta , t \right) \cdot \\&\cdot \mathbb {1}_{C}\left( \rho {\text {cos}}\theta + s {\text {sin}}\theta , \rho {\text {sin}}\theta - s {\text {cos}}\theta \right) ds , \end{aligned} \end{aligned}$$

where $r, 0\le r \le \pi$ rad/pixel is the maximum frequency and $\pi$ is the maximum bandwidth of the visual field. Furthermore, $-r \le \rho \le r, 0\le \theta < \pi$, and

7 $$\begin{aligned} \mathbb {1}_{C}(\omega _x, \omega _y) = \left\{ \begin{array}{cc} 1, &{} \text{ if } (\omega _x, \omega _y) \in C \\ 0, &{} \text{ otherwise } . \end{array} \right. \end{aligned}$$

Here, $\rho$ and $\theta$ determines the line *L* over which the integral is computed. $\rho$ is the distance (can be both positive and negative) of the line *L* from the origin, and $\theta$ is the angle the normal vector to *L* makes with the $\omega _x$-axis. *s* is the coordinate on the line *L* with the origin set to the point with the shortest distance to the origin.

The reason to use only the values of the derivative of phase over the circular domain is to consider the same maximum frequency *r* in all directions. As we will see later, this maximum frequency will determine the highest velocity that can be correctly estimated.

If motion occurs around $(x_0, y_0)$ at a velocity of ${\textbf{v}}(t) = (v_x(t), v_y(t))$, the phase gradient is approximately

$$\begin{aligned} \frac{\text {d}\phi _{x_0,y_0}}{\text {d}t}(\omega _x,\omega _y,t) = -v_x(t)\omega _x - v_y (t) \omega _y \end{aligned}$$

(see also Appendix A.2). Then,

8 $$\begin{aligned} \begin{aligned}&\left( {\mathcal {R}} \frac{\text {d}\phi _{x_0,y_0}}{\text {d}t}\right) (\rho , \theta , t) = \int _{{\mathbb {R}}} \left[ -v_x(t) \left( \rho {\text {cos}}\theta + s {\text {sin}}\theta \right) \right. \\&\quad \left. - v_y(t) \left( \rho {\text {sin}}\theta - s {\text {cos}}\theta \right) \right] \cdot \\&\quad \cdot \mathbb {1}_C \left( \rho {\text {cos}}\theta + s {\text {sin}}\theta , \rho {\text {sin}}\theta - s {\text {cos}}\theta \right) \text {d}s . \end{aligned} \end{aligned}$$

We note that since

9 $$\begin{aligned} \int _{{\mathbb {R}}} s \mathbb {1}_C \left( \rho {\text {cos}}\theta + s {\text {sin}}\theta , \rho {\text {sin}}\theta - s {\text {cos}}\theta \right) \text {d}s = 0, \end{aligned}$$

we have

10 $$\begin{aligned} \begin{aligned}&\left( {\mathcal {R}}\frac{d\phi _{kl}}{dt}\right) (\rho ,\theta ,t) = \rho \left( -v_x(t) {\text {cos}}\theta - v_y(t) {\text {sin}}\theta \right) \cdot {\mathfrak {c}}(\rho ,\theta ) \\&\quad = - \rho \sqrt{v_x^2(t)+v_y^2(t)}{\text {cos}}(\theta \\&\qquad -{\text {arctan2}}(v_y(t), v_x(t))) \cdot {\mathfrak {c}}(\rho ,\theta ) , \end{aligned} \end{aligned}$$

where

11 $$\begin{aligned} {\mathfrak {c}}(\rho , \theta ) = \int _{{\mathbb {R}}} \mathbb {1}_C\left( \rho {\text {cos}}\theta + s {\text {sin}}\theta , \rho {\text {sin}}\theta - s {\text {cos}}\theta \right) ds.\nonumber \\ \end{aligned}$$

The PMI is defined as

12 $$\begin{aligned} \text{ PMI}_{x_0,y_0}(t) = \underset{\theta \in [0,\pi )}{{\text {max}}} \int _{-r}^{r} \left| \frac{({\mathcal {R}}\frac{\text {d}\phi _{kl}}{\text {d}t})(\rho ,\theta ,t)}{{\mathfrak {c}}(\rho ,\theta )} \right| d\rho , \end{aligned}$$

with

13 $$\begin{aligned} {\hat{\theta }}_{x_0, y_0}(t) = \underset{\theta \in [0,\pi )}{{\text {argmax}}} \int _{-r}^{r} \left| \frac{({\mathcal {R}} \frac{d\phi _{x_0,y_0}}{dt})(\rho ,\theta ,t)}{{\mathfrak {c}}(\rho ,\theta )}\right| d\rho . \end{aligned}$$

If (10) holds, the PMI amounts to

14 $$\begin{aligned} \text{ PMI}_{x_0,y_0}(t)&= 2 \int _{0}^{r} \rho \sqrt{v_x^2(t)+v_y^2(t)} d\rho \nonumber \\&= r^2 \sqrt{v_x^2(t)+v_y^2(t)}. \end{aligned}$$

Thus, the PMI is proportional to the magnitude of the motion velocity if motion occurs around the point $(x_0,y_0)$. Furthermore, the angle maximizing the PMI in (12) is given by (see also Eq. (10))

15 $$\begin{aligned} {\hat{\theta }}_{x_0,y_0}(t) = {\text {arctan2}}(v_y(t), v_x(t)) {\text {modulo}} \pi . \end{aligned}$$

Therefore, ${\hat{\theta }}_{x_0, y_0}(t)$ is the angle of motion. The direction of motion along the angle ${\hat{\theta }}_{x_0,y_0}(t)$ is determined by the sign of $({\mathcal {R}}\frac{d\phi _{kl}}{dt})(\rho , {\hat{\theta }}_{x_0,y_0},t)$ for $\rho > 0$. According to (10), if this sign is $-1$, then the direction of motion is ${\hat{\theta }}_{x_0,y_0}(t)$. If this sign is 1, then the direction of motion is ${\hat{\theta }}_{x_0,y_0}(t) + \pi$.

It is important to recognize that the planar structure in $\frac{\text {d}\phi _{x_0,y_0}}{\text {d}t}$ is limited to certain frequencies, due to phase wrapping. For example, translation along the x-axis between two consecutive frames by *k* units will result in a phase shift at frequency $\omega _x$ by $k\omega _x$. If $k \omega _x > \pi$, then the detected phase change is indistinguishable from $k \omega _x$ modulo $2\pi$. This is the reason why we use only the values of the derivative of phase restricted in a circular domain. Thus, the maximum velocity of motion that can be accurately extracted is $kr \le \pi$ or $k \le \frac{\pi }{r}$ in pixels per frame, where *r* is the maximum frequency $\omega _x$ can take within the domain *C*.

In the neural circuit of early visual system of the fruit fly brain, the inputs and all processing are in the analog domain. That is, neurons communicate through graded potential values rather than spikes. Therefore, in such an analog circuit, phase aliasing does not occur.

### Intensity and contrast level sensitivity of local phase-based motion detectors

In this section, we demonstrate the effectiveness of the motion detector operating on visual stimuli with a wide range of brightness and contrast levels. We then show that its performance will be limited by how well visual stimuli are encoded in the photoreceptors.

**Fig. 2:**
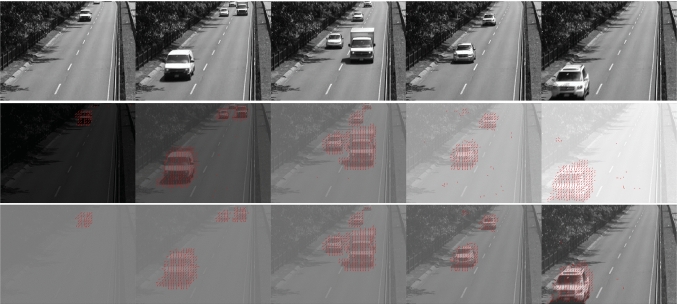
Phase-based motion detector operating on a visual stimulus under different brightness and contrast levels. (top row) The original video sequence with 8-bit pixel precision (Goyette et al. 2012). 5 representative frames of the video sequence are displayed. (2nd row) Each of the 5 frames exhibits one of the five light intensity levels that are increasing by a factor of 10 in each column. The detected motion is indicated by the red arrows. (3rd row) Each of the 5 frames exhibits one of the five contrast levels that are increasing by a factor of 2 in each column (see the text for more details). The detected motion is indicated by the red arrows

We first tested the phase-based motion detector using a video sequence of the change detection dataset (Goyette et al. 2012). Fig. 2(top row) shows 5 representative frames of the original monochrome video sequence. From this sequence, we constructed a video sequence in which the light intensity was artificially increases by a factor of 10 after every 100 frames (see Appendix B for the detailed procedure). A sample frame for each brightness level of the new video sequence is depicted in Fig. 2(2nd row). The pixels of the frames in the first and second rows of Fig. 2 are in one-to-one correspondence.

Example results of evaluating the local phase-based motion detection algorithm are shown in Fig. 2(2nd row). The red arrows indicate the motion detected at the chosen time instance.

In the next example, by increasing the Michelson contrast (Michelson 1927) of the video sequence with a fixed brightness level, we tested the performance of the local phase-based motion detector under different contrast levels. We created a video sequence in which the local contrast was artificially increased by a factor of 2 after every 100 frames (see Appendix B for the detailed procedure). A sample frame at different contrast levels for the video sequence is depicted in Fig. 2(3rd row). Red arrows indicate the motion detected at the chosen time instance.

**Fig. 3:**
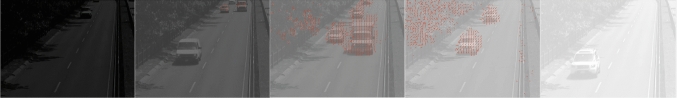
Motion detection applied onto the same video sequence as in the second row of Fig. 2 after preprocessing by a sigmoidal function. The motion detector can no longer detect motion at very low and very high brightness levels

Figure 2 demonstrates that the local phase-based motion detector can robustly detect motion emerging in visual fields with a wide range of brightness and contrast levels. The underlying assumption here is that the light sensor is ideal and has no saturation level. In reality, fly photoreceptors have a limited dynamic range. Once the stimulus is large enough, the output of a photoreceptor becomes saturated. This leaves no room for a motion detector to operate accurately. For example, the response of fly photoreceptors (and vertebrate cones) to different brightness levels typically follows a sigmoid (Sterling and Laughlin 2015). In Fig. 3 after encoding the video recording with a typical sigmoidal function with a linear range covering 2 orders of magnitudes, the motion detector can no longer detect motion robustly at brightness saturation levels.

## Modeling intensity and contrast gain control with divisive normalization processors

In order for the motion detector described in Sect. 2 to operate in a dynamically changing environment with a wide range of light intensity and contrast levels, there is a need for controlling the output range of the photoreceptors (Sterling and Laughlin 2015). In this section, we present a DNP modeling the intensity and contrast gain control at the photoreceptor/amacrine cell layer in the fruit fly lamina.

### Divisive normalization processors modeling the photoreceptor/amacrine cell layer

Photoreceptors alone (see Fig. 1) cannot achieve effective intensity and contrast gain control if they operate independently. To incorporate spatial information, neurons interconnecting photoreceptors in a neighborhood are needed. Amacrine cells are a perfect candidate. They are interneurons local to the Lamina neuropil, and their processes innervate multiple cartridges. They form reciprocal synapses with photoreceptors in these cartridges.

**Fig. 4:**
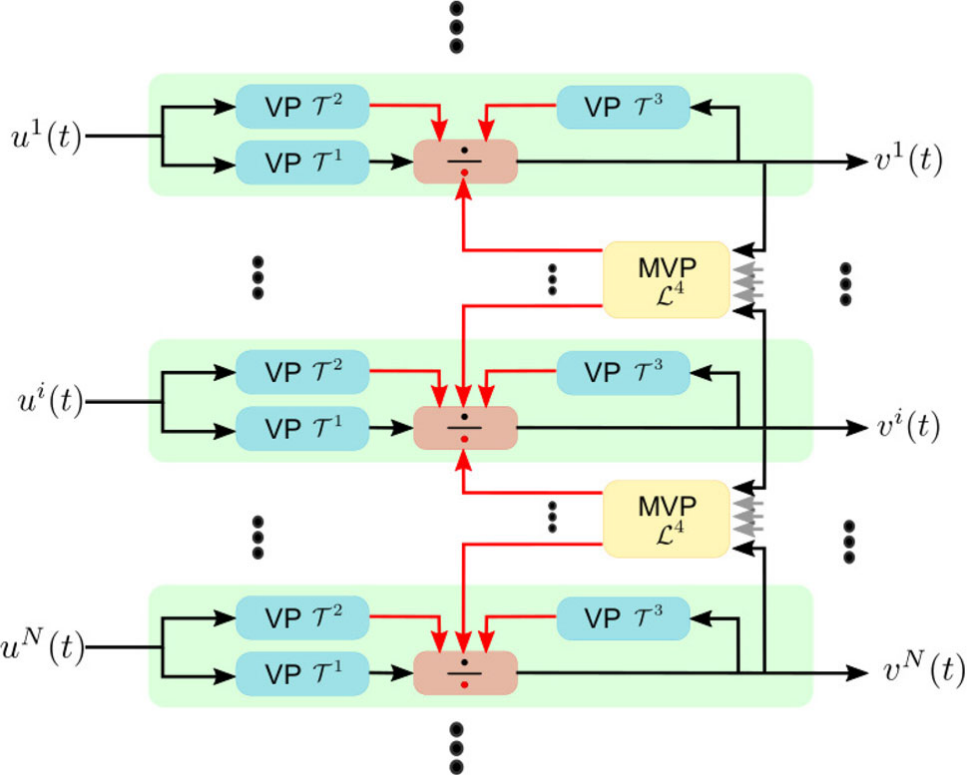
A diagram of the spatio-temporal DNP modeling the photoreceptor/amacrine cell layer. The DNP consists of *N* channels. In channel *i*, the input $u^i(t)$ is processed by two Volterra Processors (VPs), ${\mathcal {T}}^1$ and ${\mathcal {T}}^2$, and fed into a division unit. The output $v^i(t)$ is processed by a third VP ${\mathcal {T}}^3$ and fed into the same division unit. Outputs from channels in a neighbourhood are jointly processed by a Multi-input Volterra Processor (MVP) and fed into the division units of all the corresponding channels. The black arrow inputs to the division unit are passed to the numerator, and the red arrow inputs are summed in the denominator

To model the interaction of photoreceptor axon terminals and amacrine cells, we have introduced divisive normalization processors in the space-time domain (Lazar et al. 2020). Figure 4 depicts a schematic diagram of the spatio-temporal DNP modeling the photoreceptor/amacrine cell layer. Here, the spatio-temporal DNP consists of parallel temporal DNPs with the added cross-channel feedback normalization/gain control provided by the MVP blocks. The temporal DNP blocks model the local photoreceptor/amacrine cell interaction and the MVP blocks model the spatio-temporal photoreceptor/amacrine cell feedback in a restricted spatial neighborhood of the photoreceptor/amacrine cell layer.

Given the input to each of the photoreceptors $u^i(t), i = 1, 2,\ldots , N$, the output of the spatio-temporal DNP $v^i(t), i = 1, 2, \ldots , N$, satisfies the equations:

16 $$\begin{aligned} v^i(t) = \frac{{\mathcal {T}}^1 u^i}{{\mathcal {T}}^2 u^i + {\mathcal {T}}^3 v^i + {\mathcal {L}}^4{\textbf{v}}}, \end{aligned}$$

for $$i = 1, 2,\ldots , N$$, where

17 $$\begin{aligned} \left( {\mathcal {T}}^l u^i\right) (t){} & {} = b^l + \int _{{\mathbb {R}}} h^l_1(s) u^i(t-s) \text {d}s \nonumber \\{} & {} \quad + \int _{{\mathbb {R}}^2} h^l_2(s_1,s_2)\nonumber \\{} & {} \quad u^i(t-s_1)u^i(t-s_2) ds_1 ds_2, ~~ l = 1, 2, \end{aligned}$$

18 $$\begin{aligned} \left( {\mathcal {T}}^3 v^i\right) (t){} & {} = b^3 + \int _{{\mathbb {R}}} h^3_1(s) v^i(t-s) \text {d}s \nonumber \\{} & {} \quad + \int _{{\mathbb {R}}^2} h^3_2(s_1,s_2)\nonumber \\{} & {} \quad v^i(t-s_1)v^i(t-s_2) \text {d}s_1 \text {d}s_2, \end{aligned}$$

and

19 $$\begin{aligned} \begin{aligned}&({\mathcal {L}}^4{\textbf{v}})(t) = b^4 + \sum _{i=1}^N\left( \int _{{\mathbb {R}}} h^{i4}_1(s) v^i(t-s) \text {d}s \right) \\&\quad + \sum _{i=1}^N \sum _{j=1}^N \left( \int _{{\mathbb {R}}^2} h^{ij4}_2(s_1,s_2) v^i(t-s_1)v^j(t-s_2) \text {d}s_1 \text {d}s_2\right) , \end{aligned} \end{aligned}$$

with ${\textbf{v}}(t) = \left[ v^1(t), v^2(t), \ldots , v^N(t)\right] ^{T}$. Here, $b^l, l = 1,2,3,4$, are zero’th-order Volterra kernels, $h^{l}_1, l = 1,2,3$, and $h^{i4}_1, i = 1,2,\ldots ,N$, are first-order Volterra kernels, and $h^{l}_2, l = 1,2,3$, and $h^{ij4}_2, i,j = 1,2,\ldots ,N$, are second-order Volterra kernels.

In what follows, we will evaluate the I/O mapping of each DNP type, building upon the ones shown in Fig. 4. We first consider a temporal DNP block with only the feedforward terms. The diagram of the feedforward DNP blocks, shown in Fig. 4, is described by the I/O pair $(u^i,v^i)$ and

20 $$\begin{aligned} v^i(t) = \frac{{\mathcal {T}}^1 u^i}{{\mathcal {T}}^2 u^i}. \end{aligned}$$

**Fig. 5:**
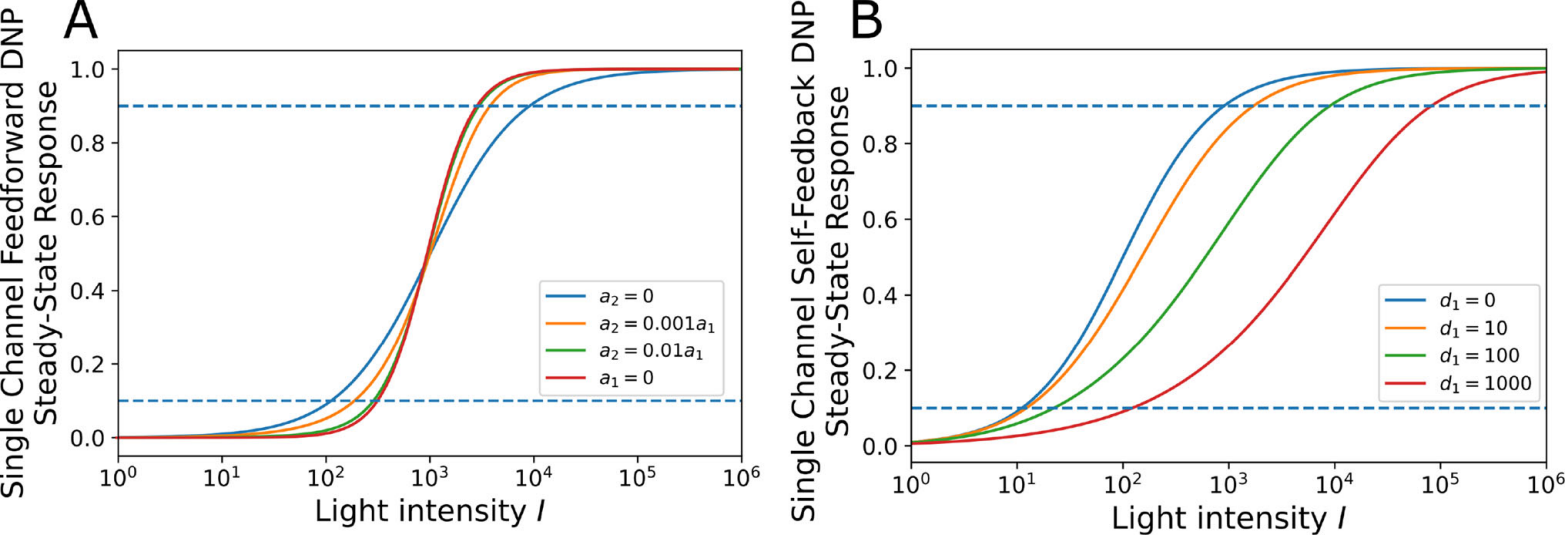
The steady-state response curve as a function of the parameters of a temporal DNP. **a** For the feedforward DNP block shown in Fig. 4, increasing the ratio between $a_2$ and $a_1$ increases the slope of the sigmoid. **b** For the DNP block with feedforward and local feedback as shown in Fig. 4, increasing the feedback strength decreases the slope of the sigmoid. Dashed lines indicate the 0.1 and 0.9 levels

For an input with constant intensity value *I*, the steady state response of the DNP is given by

21 $$\begin{aligned} v^i(I) = \frac{a_0 + a_1I + a_2 I^2}{c_0 + c_1 I + c_2 I^2}, \end{aligned}$$

where $a_0 = b^1$, $a_1 = \int _{{\mathbb {R}}} h^1_1(t)\text {d}t$, $a_2 = \int _{{\mathbb {R}}^2} h^1_2(t_1, t_2) dt_1 dt_2$ are, respectively, the DC components of the first and second-order Volterra kernels of ${\mathcal {T}}^1$, and $c_0 = b^2$, $c_1 =\int _{{\mathbb {R}}} h^2_1(t)\text {d}t$, $c_2 = \int _{{\mathbb {R}}^2} h^2_2(t_1, t_2) dt_1 dt_2$ are, respectively, the DC components of the first and second-order Volterra kernels of ${\mathcal {T}}^2$. For simplicity, to ensure that the denominator is always positive, we also assume that the coefficients $a_0, a_1, a_2, c_0, c_1, c_2$ are positive. We consider $v^i(I)$ to be normalized and to take values between 0 and 1. This can be achieved by working with $c_2 / a_2 \cdot v^i(I)$ or simply with $v^i(I)$ by setting $c_2 = a_2$. Consequently, the steady state response of a temporal feedforward DNP is a sigmoidal function of ${\text {log}}_{10}(I)$ with an output range between 0 and 1 (see also Appendix C.1). The steady-state response curve is shown in Fig. 5A for a few values of the $a_2 / a_1$ ratio. We note that the slope of the sigmoid gradually increases from a first-order Volterra kernel ($a_2=0$) to a second-order Volterra kernel ($a_1=0$). Here, by choosing $c_1\ge a_1$, we ensured that $v^i(I)$ is a monotonically increasing function (see also Appendix C.1).

By adding the local-feedback term ${\mathcal {T}}^3v^i$, we obtain the DNP block with feedforward and local feedback depicted in Fig. 4. The output of this temporal DNP can be expressed as

22 $$\begin{aligned} v^i(t) = \frac{{\mathcal {T}}^1 u^i}{{\mathcal {T}}^2 u^i + {\mathcal {T}}^3 v^i}, i = 1, 2, \ldots , N. \end{aligned}$$

Its steady-state response is given by

23 $$\begin{aligned} v^i(I) = \left\{ \begin{array}{cc} \frac{-C + \sqrt{C^2 - 4d_1A}}{2 d_1} &{} \text{ if } d_2 = 0, \\ -\frac{1}{3d_2}\left( d_1 + D \frac{-1+\sqrt{-3}}{2} + \frac{\Delta _0}{D\frac{-1+\sqrt{-3}}{2}} \right) &{} \text{ if } d_2 \ne 0, \Delta > 0, \\ -\frac{1}{3d_2}\left( d_1 + D + \frac{\Delta _0}{D}\right) &{} \text{ if } d_2 \ne 0, \Delta \le 0 \end{array} \right. \nonumber \\ \end{aligned}$$

where

24 $$\begin{aligned} A&= -(a_0+a_1I + a_2 I^2), \end{aligned}$$

25 $$\begin{aligned} C&= (c_0+d_0+c_1I + c_2I^2),\end{aligned}$$

26 $$\begin{aligned} D&= \root 3 \of { \frac{\Delta _1 + \sqrt{-\Delta }}{2} }, \end{aligned}$$

27 $$\begin{aligned} \Delta&= \frac{4\Delta _0^3 - \Delta _1^2}{27d^2_2}, \end{aligned}$$

28 $$\begin{aligned} \Delta _0&= d_1^2 - 3d_2C, \end{aligned}$$

29 $$\begin{aligned} \Delta _1&= 2d_1^3 - 9d_2d_1C + 27d_2^2A, \end{aligned}$$

with $d_0 = b^3$, $d_1 = \int _{{\mathbb {R}}} h^{3}_1(s) ds$ and $d_2 = \int _{{\mathbb {R}}^2} h^{3}_2(s_1, s_2) ds_1ds_2$. Again, we assume that the coefficients $d_i > 0, i=1,2,3$. The effect of the feedback on the steady-state response is depicted in Fig. 5B. By increasing $d_1$ (and/or $d_2$), the slope of the sigmoid decreases.

Combined with the ratio between $a_1$ and $a_2$, we see that a temporal DNP with feedforward and local feedback exhibits a range of gradients of the sigmoid for different parameter choices. This key feature underlies the contrast gain control mechanism exerted by DNPs.

**Fig. 6:**
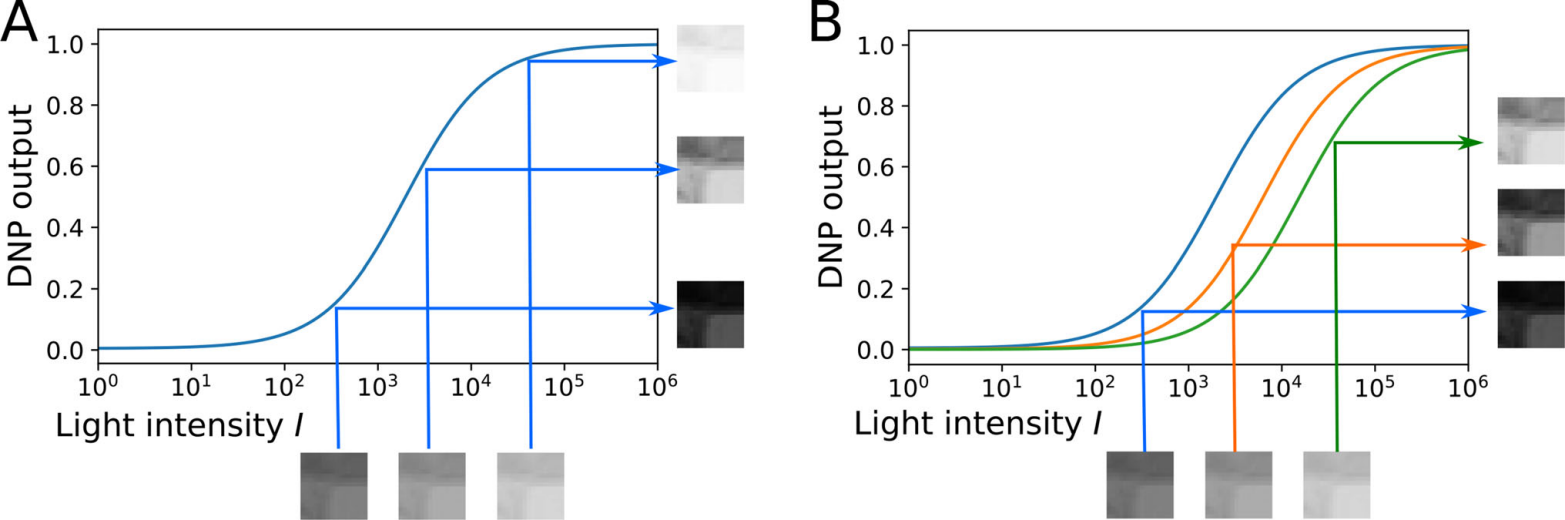
The effect of the MVP feedback. **A** The steady-state response curve of the DNP without global MVP feedback. The single curve maps three $16\times 16$ image patches at different brightness levels (bottom) into the output on the right. **B** With the MVP, the steady-state response curve shifts to the orange curve when mapping the image patch in the middle and to the green curve when mapping the image patch on the right. This allows the DNP to map a larger range of light intensity values onto the linear part the response curve, increasing the local contrast

Finally, the output of each channel also depends on the global feedback generated by the MVPs. The ${\mathcal {L}}^4{\textbf{v}}$ term in the denominator of Eq. (16) leads to a shift of the steady-state response curve to the left or right (see Fig. 6 and the discussion below) depending on the strength of the global feedback, that in turn, depends on the inputs to all channels.

**Fig. 7:**
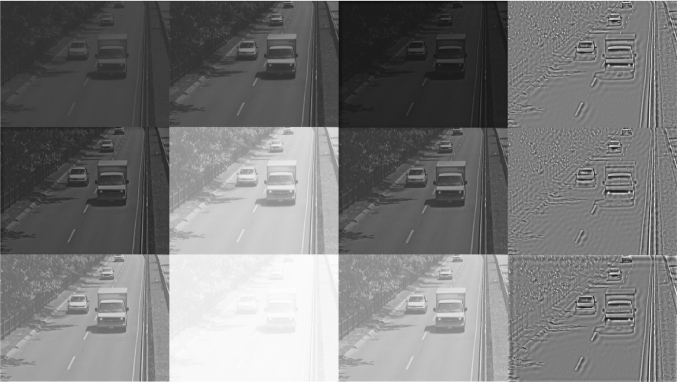
Comparison between DNP processed images at 3 different brightness levels. (1st column) Input image. (2nd column) Output of the DNP without global feedback. (3rd column) Output of the DNP with global feedback. (4th column) Output of the DNP with adaptive feedback. The image in each consecutive row has its brightness scaled by a factor of 10

In Fig. 6, we depict the mapping of a $16\times 16$ image block at three different light intensity levels, with a factor of 10 between each two consecutive levels. They are shown under the *I*-axis in both Fig. 6A and B. Without the MVP feedback term, the steady-state response curve of each pixel is fixed on the blue continuous curve for all three light intensity levels (see Fig. 6A). With the MVP, the mapping shifts to the orange curve for the image patch scaled by a factor of 10, and to the green curve for the image patch scaled by a factor of 100. The respective output of the DNPs without or with MVP are shown on the right of Fig. 6A and B, respectively. Figure 7(2nd and 3rd column) shows the outputs of the DNP with the same parameters without and with MVP, respectively, processing an entire image at 3 different brightness levels.

### Adaptive feedback intensity gain control

From the analysis of the previous section, if the input is scaled up by a factor, the output of ${\mathcal {L}}^4{\textbf{v}}$ must be scaled up by approximately the same amount. However, since the value of the output is restricted between 0 and 1, scaling up the MVP feedback is necessary.

By noticing the role that the MVP plays in the I/O curve of the DNP, we devised a DNP with a dynamic feedback term controlling the I/O curve:

30 $$\begin{aligned} v^i(t) = \frac{{\mathcal {T}}^1 u^i}{{\mathcal {T}}^2 u^i + {\mathcal {T}}^3 v^i + w}, \end{aligned}$$

where

31 $$\begin{aligned} \frac{\text {d}w}{\text {d}t} = \alpha \left[ {\mathcal {L}}^4{\textbf{v}}(t) - 0.5(b^4+r_1 + r_2)\right] , \end{aligned}$$

with $\alpha >0$, $r_1 = \sum _{i=1}^N \int _{{\mathbb {R}}} h^{i4}(t) dt$ and $r_2 = \sum _{i=1}^N\sum _{j=1}^N \int _{{\mathbb {R}}^2} h^{ij4}(t_1, t_2) dt_1 dt_2$. Eq. (31) centers the steady-state the outputs of the DNP channels to a mean of 0.5, the middle point of the sigmoid curve. Consequently, the output of the photoreceptor will be distributed on both sides of the center of the sigmoid and will mostly operate in the linear range of the sigmoid.

**Fig. 8:**
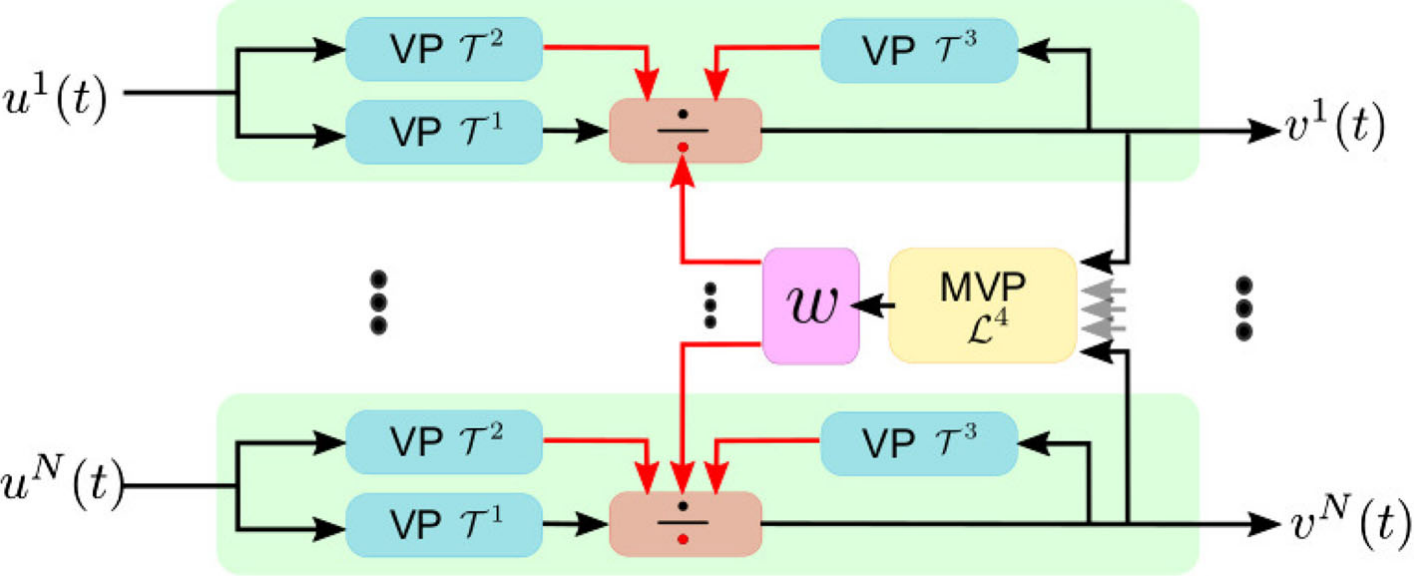
The schematic diagram of the adaptive feedback DNP model with an additional processor *w* in the feedback loop. See also the notation in Fig. 4–here, for simplicity, only two channels and one MVP are depicted

Figure 8 shows a schematic diagram of the DNP introduced above. We note that Eq. (31) suggests an additional processor in the feedback loop. Such a feedback loop may well be implemented in the molecular domain. For example, photoreceptors operate at a baseline intracellular calcium concentration level. Calcium accumulation can cause this baseline to increase by a factor of more than 1,000 and thereby shift the response curve of the photoreceptor (Song et al. 2012; Sterling and Laughlin 2015).

Figure 7(4th column) displays the output of the adaptive feedback DNP for the inputs in Fig. 7(1st column). We note that the output of this DNP is approximately the same for all 3 inputs with different brightness levels. The DNP also enhances the contrast at the edges.

## Evaluation of motion detection with gain control preprocessing

In this section, we combine the results in the previous two sections, and model the overall motion detection of the fruit fly early visual system consisting of the retina, lamina and medulla (see Fig. 1A) with a cascade of two DNP processing blocks, as shown in Fig. 1B. The DNP in the first block models the intensity and contrast gain control at the photoreceptors/amacrine cells layer. The second block consists of a DNP for detecting phase changes followed by a phase-based motion detector. This block models the elementary motion detection by the fruit fly medulla/lobula T4/T5 neurons using local phase information.

### Evaluation of motion detection with DNPs

Existing benchmark optic flow datasets are not suitable for evaluating phase-base motion detection algorithms. They either have very few frames (Baker et al. 2011; Geiger et al. 2012; Dosovitskiy et al. 2015) or have a too large displacement between frames (Butler et al. 2012; Mayer et al. 2016) to allow phase changes to be correctly estimated.

In what follows, we will evaluate the phase-based motion detector and compare its performance with other algorithms using videos captured by the authors in a local park. We note that inputs to the fly photoreceptors are analog, and the visual processing in the early visual system of the fruit fly brain is also “analog" as well. To approximate the analog visual world, we captured videos at the highest mobile phone frame rate.

#### Qualitative evaluation of phase-based motion detectors

To evaluate the motion detector performance, we used two types of video recordings. The first type were shot at 240 frames per second (fps) using a Samsung S23 Plus mobile phone at Full High Definition (FHD) $1920\times 1080$ resolution. Each color frame was converted to grey-scale luminance and resized to $480\times 270$ before fed into the phase-based motion detector and other motion detection algorithms for comparison. The input pixel values were already gamma corrected when the video sequence was shot with a bit-depth of 8 bit.

**Fig. 9:**
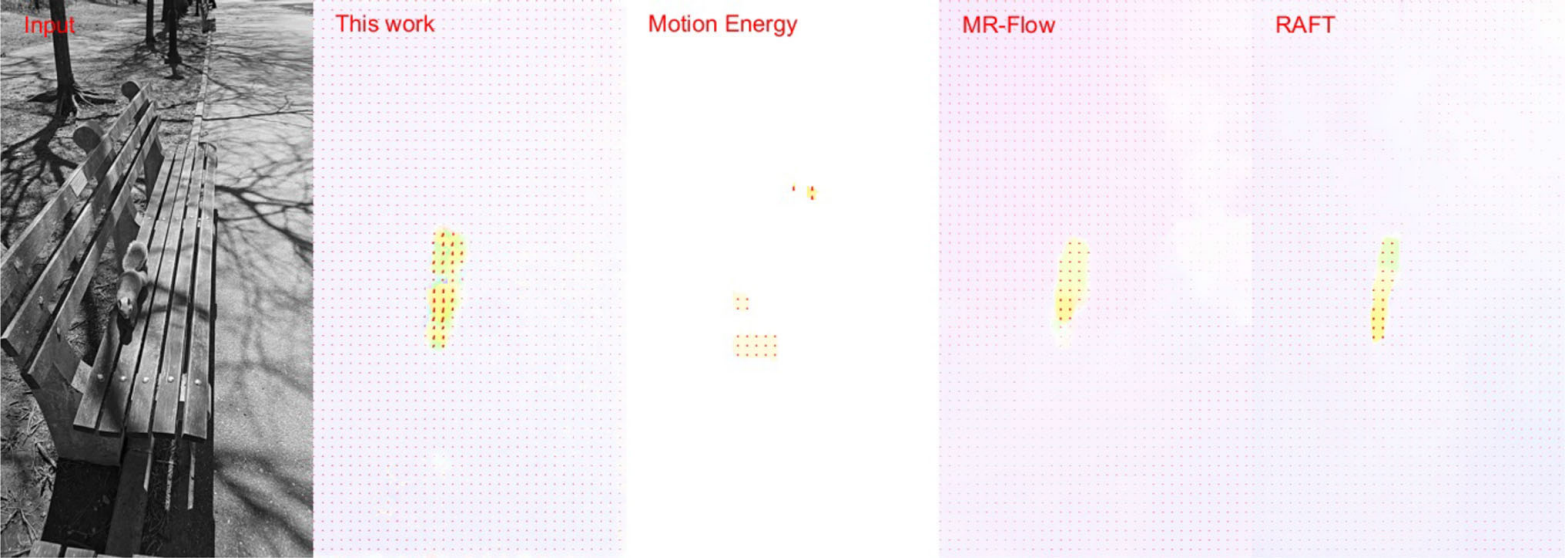
Comparison of motion detected in a visual sequence shot at 240 fps. (1st column) A sample frame of a 240 fps visual sequence. (2nd column) Motion detected by the phase-based motion detector. (3rd column) Motion detected by the motion energy algorithm (Shi and Luo 2018). (4th column) Motion detected by the MR-Flow algorithm (Wulff et al. 2017). (5th column) Motion detected by the RAFT algorithm (Teed and Deng 2020). Direction of detected motion is indicated by the common color coding convention (Baker et al. 2011) as well as by arrows on a sparser grid. See Video S1 for the full video

Since there was no ground truth for motion in these visual sequences, we visually compared the performance of motion detection using (1) the phase-based motion detection algorithm, (2) the algorithm of Shi and Luo (2018) based on the motion energy model inspired by the mammalian brain, and (3) the MR-Flow algorithm (Wulff et al. 2017) and (4) the top performing RAFT algorithm in the MPI-Sintel benchmark (Butler et al. 2012). Note that the more recent RAFT algorithm is based on artificial neural networks (Shah and Xuezhi 2021). Motion detection was performed at every other pixel using method (1), on frames subsampled to $240\times 135$ by method (2), and on the full $480\times 270$ frames by methods (3) and (4). Therefore, the detected motion using methods (3) and (4) had four times the density of the motion detected by methods (1) and (2). All models mentioned above were used in all experiments with the same set of parameters.

For methods (1), (2) and (3) we used consecutive frames when performing motion detection. For method (4), however, we did not obtain any meaningful output due to the small displacement between frames. Therefore, the optic flow algorithm was computed using frames that are 1/30 second apart.

Figure 9(1st column) shows a sample frame of the natural visual sequence with a squirrel moving downward on a bench. A small background movement due to hand movement while shooting the video is also discernible. The phase-based motion detector captured both the movement of the squirrel and the background (2nd column). Its performance is comparable to the MR-Flow (4th column) and the RAFT algorithms (5th column), if not better, and much better than that of the motion energy model (3rd column). The full video is available as Video S1 in the Supplementary Materials. Additional examples can be found in Video S2, 3.

**Table 1:** Comparison of the processing speed of different motion detection algorithms

Algorithm	Environment	Processing speed (fps)
Phase-based (this work)	$1\times$ NVIDIA V100 GPU (Python)	170
Motion energy model (Shi and Luo 2018)	$1\times$ NVIDIA V100 GPU (Python)	480
MR-Flow (Wulff et al. 2017)	$2\times$ Intel Xeon Gold 5120 CPU (14 cores @ 2.2GHz) (Python)	0.013
RAFT (Shah and Xuezhi 2021)	$1\times$ NVIDIA V100 GPU (Python)	5.33

The execution of phase-based motion detector, the MR-Flow algorithm and the RAFT algorithm was frame by frame, while the motion energy model is executed in batches of frames. Therefore, the actual real-time processing speed may be lower for the motion energy model

Table 1 specifies the code execution environment of the 4 different algorithms. The processing of the phase-based motion detector is highly efficient on GPUs and can process 170 frames per second. While the motion energy model can be executed at 480 frames per second, we note that the algorithm jointly processes batches of frames. Since the motion energy model requires temporal filtering, it will be slowed down when implemented frame by frame in real-time applications.

The second type of video sequences were shot at 60 fps using the same mobile phone at FHD resolution. The raw sensor data had 13 bit bit-depth. We used only the green color channel of the raw frames to ensure that the pixel values are approximately linear with respect to the light intensity of the visual scenes. Each frame was then resized to $480\times 270$ before fed into motion detectors. These videos exhibit rapid changes in light intensity and contrast across the scene.

**Fig. 10:**
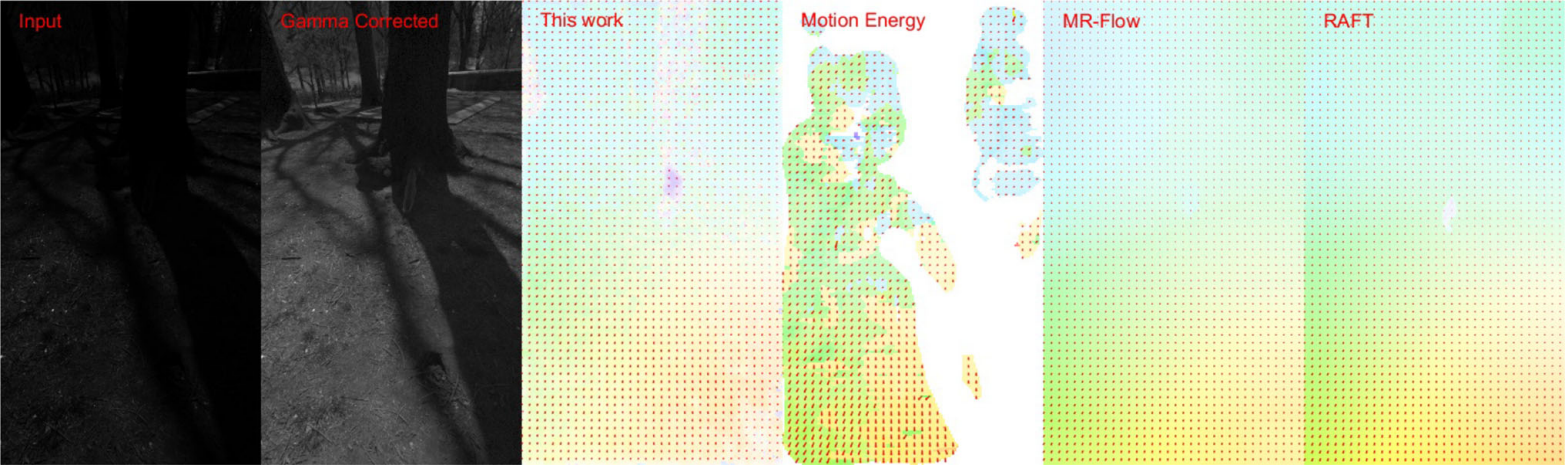
Comparison of motion detected in a “raw” visual sequence shot at 60 fps. (1st column) A sample “raw” frame of a 60 fps visual sequence. Pixel values are proportional to light intensity. (2nd column) The video frame with gamma correction of 2.2. (3rd column) Motion detected by the phase-based motion detector. (4th column) Motion detected by the motion energy algorithm (Shi and Luo 2018). (5th column) Motion detected by the MR-Flow algorithm (Wulff et al. 2017). (6th column) Motion detected by the RAFT algorithm (Shah and Xuezhi 2021). Direction of detected motion is indicated by the common color coding convention (Baker et al. 2011) as well as by arrows on a sparser grid. See Video S4 for full video

Figure 10(1st column) shows a sample frame with a squirrel moving up on a tree trunk in the shadow with the rest of the scene exposed to direct sunlight. To better display the input, Fig. 10(2nd column) depicts the same frame with a gamma correction of 2.2. The phase-based motion detector (3rd column) successfully detected both movement of the squirrel and the background. The motion energy model (4th column) detected the background movement (which is larger than in Fig. 9, but fails to detect the squirrel’s motion under the shadow. The MR-Flow algorithm (5th column) and the RAFT algorithm (6th column) provide a smoother background motion but fails to detect the moving squirrel. This is likely due to the fact that the video frames fed into the MR-Flow algorithm had only 8 bit bit-depth and did not have enough precision to discern the image of the squirrel under the shadow. The full video is available as Video S4 in the Supplementary Materials. Additional videos can be found in Video S5, 6.

The results presented so far indicate that the phase-based motion detector performs well under natural light conditions that can significantly vary in the same scene. In Supplementary Video S7–9, we show how the phase-based motion detector performs on video sequences that are subject to additive white Gaussian noise with SNRs ranging from 30 dB to 10 dB. Note that the area with lower contrast is disproportionally affected by noise.

#### Quantitative evaluation of phase-based motion detectors

**Fig. 11:**
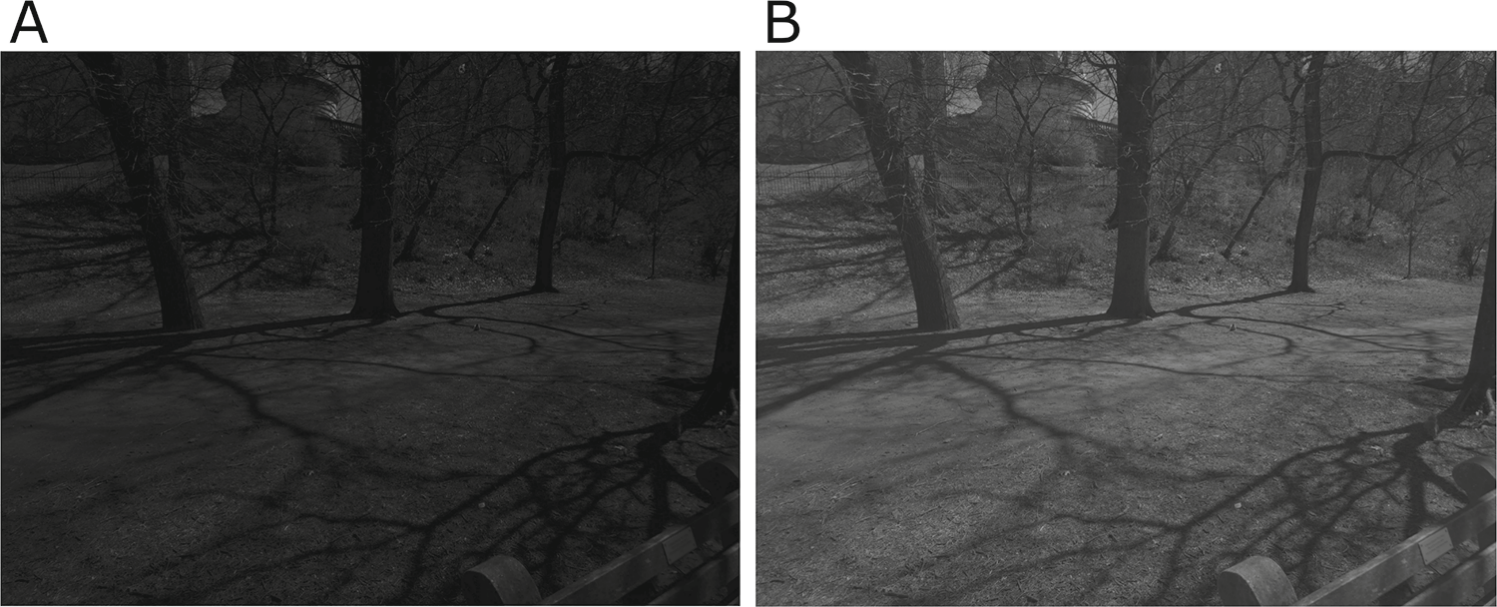
Sample raw images that are used to generate full screen motion with ground truth direction

In order to evaluate the phase-based motion detector quantitatively, we used the same mobile phone to shoot raw images at a bit-depth of 14 bits and a resolution of $8000\times 6000$. We again only used the green color channel from these raw images. A sample image and its gamma corrected version are shown in Fig. 11.

**Fig. 12:**
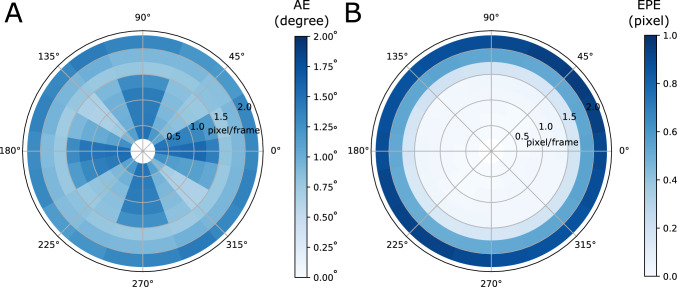
Average **A** angular error and **B** end-point error of motion detected by the phase-based motion detector. The errors are provided in a polar plot. Each angle represents the error of ground-truth motion moving in the direction indicated by the angle. Each radius shows the motion with speed at 0.25 to 2.0 pixel per frame with an 0.25 increment

For each scene, we chose a $256\times 256$ window and moved the window by a specified number of pixel per frame (from 0.25 to 2 with an increment of 0.25), similar to the approach used in Shi and Luo (2018). For speeds of a fractional pixel per frame, we used a cubic spline interpolation. We then obtained a series of video sequences with ground-truth motion direction and tested how well the phase-based motion detector can detect motion at every other pixel. In Fig. 12A, we show the average angular error (AE) in a polar plot for every tested velocity and angle. AE was largely kept under $2^{\circ }$, with an overall average of $1.09^{\circ }$. Figure 12B shows the end-point error (EPE). The EPE was largely under 0.2 pixels for speeds up to 1.5 pixel/second. We used a circular domain with $r = \frac{5}{8}\pi$, expecting that phase aliasing would occur at velocities larger than 1.6 pixel/frame. As predicted, EPE in Fig. 12B is much larger for motion velocities above 1.5.

### Evaluation of the entire DNP cascade

**Fig. 13:**
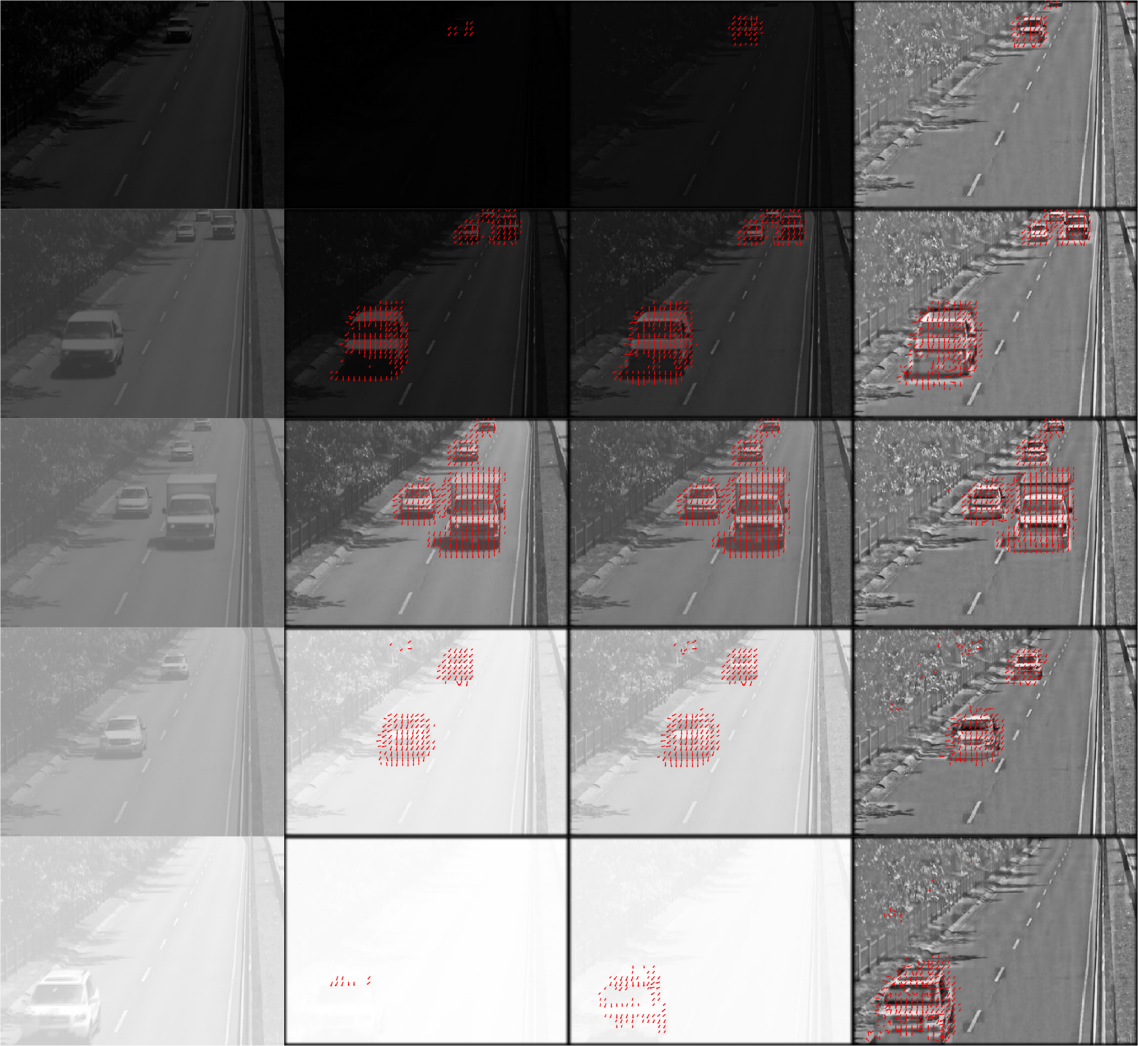
Evaluation of motion detection using a cascade of two DNPs. The video sequence has 500 frames. The brightness of the video increases by a factor of 10 after every 100 frames. The resulting video sequence has brightness that spans 5 orders of magnitude. (1st column) A typical frame at a different brightness level in each row. (2nd column) DNP output without the MVP feedback block. Red arrows indicate the motion detected from the DNP output. The motion detector cannot robustly detect motion under very low (first row) or very high (last row) brightness conditions. (3rd column) DNP output with the MVP feedback block. Red arrows indicate the motion detected from the DNP output. The output of the DNP with MVP feedback is slightly less saturated than the output of DNP without the MVP feedback. The phase-based motion detector can pick up the subtle differences after the DNP processes and detects motion. (4th column) The output of the adaptive feedback DNP. Red arrows indicate the motion detected. The output of this DNP is mostly invariant of the input brightness level. See also Supplementary Video S10

In Fig. 13, we evaluate the motion detection in a dynamically changing environments scenario with light intensity ranging over 5 orders of magnitude. Figure 13 shows results of motion detection for a video sequence processed by, respectively, the DNP without the MVP feedback, with the MVP feedback, and with the MVP adaptive feedback blocks. The brightness of each video sequence increases by a factor of 10 after every 100 frames. A typical frame at each level of brightness is shown for each video sequence in one of the 5 rows in the first column.

In the second column, we show the responses of the DNP without the MVP feedback block. The motion detected is shown in red arrows. We can see that the phase-based motion detector fails to detect motion in the lowest and highest brightness levels, since these responses are saturated.

In the third column, we show the responses of the DNP with MVP feedback block. The motion detected from these responses is shown with red arrows. The DNP outputs are less saturated and the phase-based motion detector is able to robustly detect motion.

In the fourth column, we show the responses of the DNP described in Sect. 3.2. These responses are largely invariant to the input brightness levels. The phase-based motion detector can robustly detect motion from these outputs.

The videos corresponding to Fig. 13 can be found in Supplementary Video S10. Additional video recordings are available in Supplementary Video S11–15. The parameters for testing all these video sequences were kept the same. Therefore, the outputs of the intensity and contrast gain control DNP looks drastically different from that of the DNP without the MVP block and the DNP with the global feedback block. The outputs are in a similar range when using the DNP with the adaptive feedback block across all videos. In most of the tests, however, the final motion detector responses were similar across the three configurations. This is because the phase-based motion detector can still detect motion even if the output of the first DNP has lower contrast, as shown in Fig. 2. However, we expect that motion detection will be more accurate when using the DNP with adaptive feedback.

## Discussion

The early visual system of the fruit fly, as the vertebrate retina, exhibits highly nonlinear processing of visual information in the analog domain. In this paper, we offered further insights into a class of analog nonlinear circuits, called the divisive normalization processors, first proposed in Lazar et al. (2020), and the roles they can play in modeling early visual system of the fruit fly. We demonstrated that two cascaded DNPs can effectively model the robust motion detection capability of the early visual system in the fruit fly. The two stages of DNPs, as schematically shown in Fig. 1B, implement intensity/contrast gain control and elementary motion detection, respectively.

We first introduced a feedforward DNP underlying the phase-based motion detector modeling motion detection in the fruit fly. The DNP modeling of the motion detection circuit allow us to directly relate the phase-based motion detector with the classical models of motion detection, such as the Reichardt motion detector and the motion energy model. We have extended our previous approach and developed a method of estimating both direction and velocity magnitude of motion and provided the condition under which the estimation of velocity is accurate. The performance of the motion detection circuit was quantitatively validated and extensively compared to other motion detection algorithms, such as those based on the motion energy model, as well as optic flow algorithms.

Currently many hardware implementations of bioinspired visual motion detection are based on the motion energy model of the mammalian brain (Orchard and Etienne-Cummings 2014; Shi and Luo 2018). They require a preprocessing stage to compensate for large luminance/contrast variation. For example, in Shi and Luo (2018), a threshold is first applied onto each filtered frame to enhance and normalize contrast. However, the threshold value depends on the values of luminance and contrast and a fixed threshold does not work well under all conditions. On the contrary, our model works robustly under widely different intensity and contrast environmental conditions using throughout the same set of parameters .

As demonstrated in the supplementary videos, the proposed motion detector is very effective on a range of motion velocities. In contrast, at very low speed, the optic flow algorithm we tested missed the movement of small objects whereas the phase-based motion detector was able to detect them. However, since the phase-based motion detector only processes local information, it inevitably cannot detect large displacements between frames as extensively present in most of the standard optic flow benchmarks that typically feature low frame rates. The same also applies to most of the models based on localized spatial filters such as the Gabor filters, including those derived from the motion energy model. While using additional processing after the elementary motion detection stage can be used to combine local motion information across space, frame-based processing does not appear to be a natural solution for living organisms.

As discussed in many of the examples, the phase-based motion detector takes advantage of very high data rates, possibly approaching the analog domain. Note that the fly early visual system processes visual scenes in the analog domain. Thus, our model suggests a possible reason why processing in both the early visual system of the fruit fly and the retina of vertebrates is analog. That is, the analog processing is necessary to accurately extract motion in the early visual systems. Hence, it is of interest to implement this algorithm in analog neuromorphic hardware (Stocker 2006) in the future.

While recent advances in optic flow algorithms based on artificial neural networks has led in benchmark tests to high accuracy in motion estimation, the amount of data required for training is still very high. Moreover, although deep learning has significantly improved the run time over traditional optic flow methods, they are still far from achieving real-time processing capability. In contrast, the phase-based motion detection algorithm requires no training and can process video sequences in real-time. The examples in the supplementary videos show that, under natural environments the phase-based motion detector can detect motion that is undetectable by algorithms based on deep learning. Most likely the datasets used to train these models did not contain such examples and the trained model cannot generalize robustly under these conditions.

We showed that the performance of the DNP modeling the very first stage of visual processing, i.e., the photoreceptor/amacrine cell layer, was critical for subsequent motion detection in dynamic environments where the light intensity can vary by orders of magnitudes. We provided a detailed characterization of the I/O of this class of DNPs with respect to different parameter choices, and gave insights into how they perform intensity and contrast gain control (Lazar et al. 2020).

Furthermore, we advanced here a DNP architecture with an adaptive feedback MVP block. We showed that the response of the DNP with adaptive MVP feedback is largely independent of the background light intensity levels in the visual scenes. Due to its adaptive nature, the circuit is robust to variation of parameters.

While the structure of DNP modeling the photoreceptor/amacrine cell layer directly corresponds to the anatomical structure of the neural circuit, as depicted in Fig. 1A and B, the phase-based motion detection circuit is more abstract. Notably, the fly motion detection circuit is separated into ON and OFF pathways. In the ON pathway (see Fig. 1 (in yellow)), visual motion resulting in a brightness increase is detected. Visual motion that causes a brightness decrease is detected in the OFF pathway (see Fig. 1 (in purple)). It has been shown that a full Reichardt motion detector can be subdivided into a two-quadrant model (Eichner et al. 2011) where one quadrant corresponds to the ON pathway processing brightness increases and the other corresponds to the OFF pathway processing brightness decreases. Mapping the proposed motion detection circuit into these pathways is of future interest.

Overall, the work presented here demonstrated the power of DNPs in modeling computation arising in visual processing circuits of the fly brain. As fly visual circuits that are involved in other visual tasks have similar connectivity features such as extensive feedback loops, we expect that divisive normalization can serve as a key computational building block in visual processing in the bee/ zebrafish vision systems and the retina of vertebrates (Sanes and Zipursky 2010). Additional types of DNPs and further understanding of their properties will shed light on employing DNP-based algorithms in computer vision.

### Supplementary Information

Below is the link to the electronic supplementary material.

Supplementary file 1 (mp4 4060 KB)

Supplementary file 2 (mp4 13525 KB)

Supplementary file 3 (mp4 6079 KB)

Supplementary file 4 (mp4 7717 KB)

Supplementary file 5 (mp4 3106 KB)

Supplementary file 6 (mp4 12947 KB)

Supplementary file 7 (mp4 76942 KB)

Supplementary file 8 (mp4 202035 KB)

Supplementary file 9 (mp4 66535 KB)

Supplementary file 10 (mp4 41905 KB)

Supplementary file 11 (mp4 16994 KB)

Supplementary file 12 (mp4 68320 KB)

Supplementary file 13 (mp4 50543 KB)

Supplementary file 14 (mp4 64140 KB)

Supplementary file 15 (mp4 28515 KB)

Supplementary file 16 (pdf 477 KB)

## Figures and Tables

**Fig. 14:**
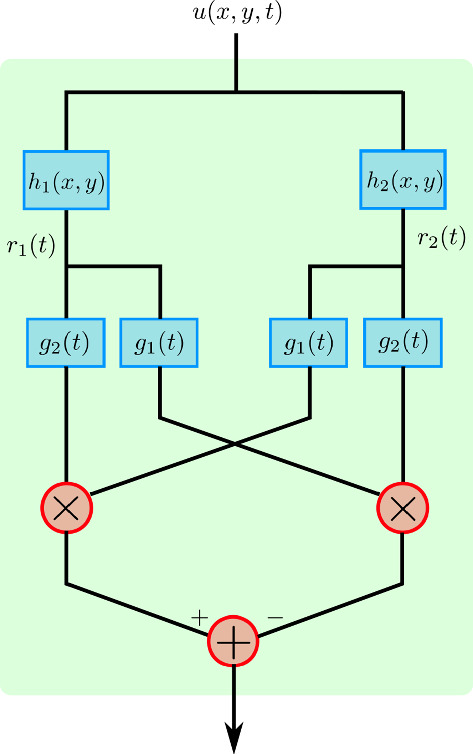
A schematic diagram of the *elaborated* Reichardt motion detector

**Fig. 15:**
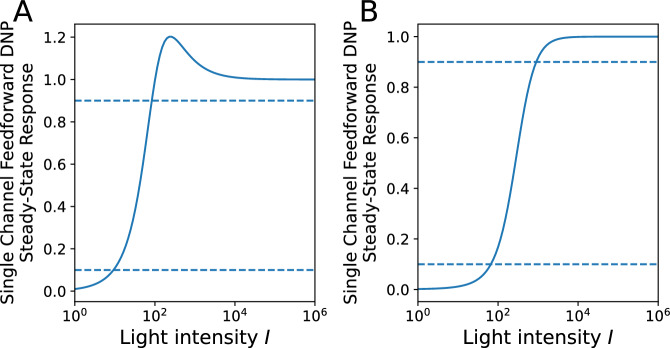
Steady-state responses of feedforward DNPs exhibit two different characteristics. **A** The steady-state response of a feedforward DNP with “overshoot" response. The following parameters were used: $a_0=1, a_1=1,000, a_2 = 10, c_0 = 100,000, c_1 = 10, c_2 = 10$. **B** Monotonically increasing steady-state response of a feedforward DNP. The following parameters were used: $a_0=10, a_1=10, a_2 = 0.1, c_0 = 100,000, c_1 = 10, c_2 = 0.1$

**Fig. 16:**
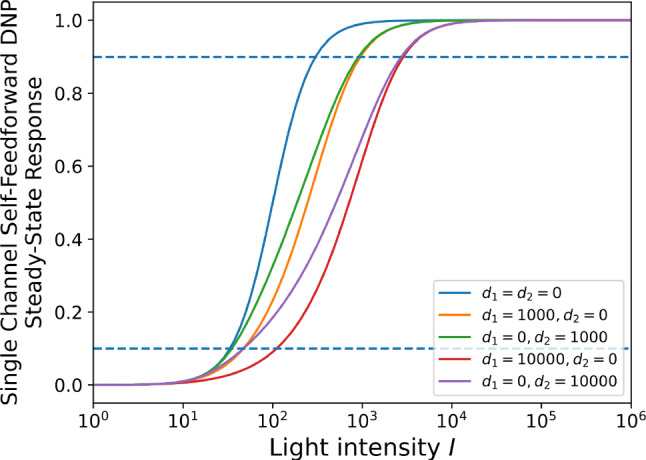
Effect of $d_1$ and $d_2$ on the steady-state response of single-channel feedback DNP. The DNP is constructed with the following parameters: $a_0 = 0, a_1 = 0, a_2 = 0.01, c_0 = 100, c_1 = 0, c_2 = 0.01$

## Models of elementary motion detectors in fruit flies

We briefly review in Appendix A.1 the structure of the elementary motion detectors and in Appendix A.2, the structure of the local phase-based motion detector.

### Reichardt motion detector

Modeling elementary motion detection circuits in fruit flies has been strongly influenced by the classical work of Hassenstein and Reichardt (1956) and Barlow and Levick (1965). Figure 14 depicts the *elaborated* Reichardt motion detector of van Santen and Sperling (1985). With properly chosen parameters, this motion detector is equivalent to the motion energy detector of Adelson and Bergen (1985) modeling direction sensitive complex cells in the mammalian visual cortex.

The Reichardt motion detector can explain many optomotor responses (Borst 2014). Electrophysiological observations of lobula plate tangential cells located downstream of these elementary motion detectors are sensitive to wide-field motion. They are, however, extremely sensitive as well to the overall brightness and contrast level of visual scenes. Since light intensity during day and night may vary by at least 6 orders of magnitude (Cronin et al. 2014), the Reichardt detector, by itself, cannot match the robustness of the *Drosophila* motion detection circuits.

An schematic diagram of the *elaborated* Reichardt motion detector is shown in Fig. 14. The output *v*(*t*), a functional of the input stimulus *u*(*x*, *y*, *t*), can be written as

32 $$\begin{aligned} \begin{aligned} v(t) = \int _{{\mathbb {R}}^6}&h_1(x_1,y_1) h_2(x_2,y_2) \left[ g_2(s_1) g_1(s_2) - g_1(s_1) g_2(s_2) \right] \cdot \\&u(x_1,y_1,t-s_1) u(x_2, y_2, t-s_2) \text {d}x_1\text {d}y_1 \text {d}s_1 \text {d}x_2\text {d}y_2 \text {d}s_2 , \end{aligned} \end{aligned}$$

where $h_1, h_2$ denote the two spatial filters and $g_1, g_2$ denote the two temporal filters shown in Fig. 14. Note that the output of the *elaborated* Reichardt motion detector is a quadratic function of the input.

### Local phase-based motion detector

In Lazar et al. (2016), we proposed a motion detector based on local phase information. With processing in the phase domain, the operation of the motion detector does not depend on the scale of the Fourier amplitude of the input stimuli. In what follows, we provide a brief overview of the local phase-based motion detector.

The local phase-based motion detector consists of 2 staged circuits, for detecting motion surrounding a reference point in the visual field. In the first stage, the local phase (defined below) and its time derivative are extracted. In the second stage, the extracted phase is processed to indicate the presence and the direction of motion. This two-stage detector can be repeated in parallel for any number of reference points covering the entire visual field.

If ${\textbf{v}} = \left( v_x(t), v_y(t)\right)$ is the instantaneous motion velocity pair, the translated signal considered here amounts to,

33 $$\begin{aligned} u(x,y,t) = u(x-s_x(t), y-s_y(t), 0), \end{aligned}$$

where

34 $$\begin{aligned} s_x(t)= & {} \int _0^t v_x(s)\text {d}s, \end{aligned}$$

35 $$\begin{aligned} s_y(t)= & {} \int _0^t v_y(s)\text {d}s. \end{aligned}$$

Consequently, the motion velocity and the time derivative of the phase satisfy the differential equations

36 $$\begin{aligned} \frac{\text {d}\phi _{x_0,y_0}}{\text {d}t}(\omega _x, \omega _y, t){} & {} = -\frac{\text {d} s_x(t)}{\text {d}t} \omega _x - \frac{d s_y(t)}{dt} \omega _y \nonumber \\{} & {} \quad + {\mathfrak {v}}_{x_0,y_0}(\omega _x, \omega _y, t), \end{aligned}$$

for all $(\omega _x, \omega _y) \in {\mathbb {R}}^2$ with the initial condition $\phi _{x_0,y_0}(\omega _x, \omega _y, 0)$, where ${\mathfrak {v}}$ is assumed to be a small term (Lazar et al. 2016).

### Definition of ${\text {arctan2}}$

37 $$\begin{aligned} {\text {arctan2}}(y, x) = \left\{ \begin{array}{cc} {\text {arctan}}\left( \frac{y}{x}\right) &{} \text{ if } x>0, \\ {\text {arctan}}\left( \frac{y}{x}\right) +\pi &{} \text{ if } x<0 \text{ and } y\ge 0, \\ {\text {arctan}}\left( \frac{y}{x}\right) -\pi &{} \text{ if } x<0 \text{ and } y< 0, \\ \frac{\pi }{2} &{} \text{ if } x= 0 \text{ and } y > 0, \\ -\frac{\pi }{2} &{} \text{ if } x = 0 \text{ and } y < 0, \\ \text{ undefined } &{} \text{ if } x = 0 \text{ and } y = 0, \end{array} \right. \nonumber \\ \end{aligned}$$

## Constructing video sequences with different light intensity and contrast levels

Here we provide details for constructing visual scenes with different light intensity and contrast levels.

### Constructing video sequences with different light intensity levels

Each pixel of the original video sequence had 8-bit precision. We note that the pixel values were already gamma corrected during recording. Prior to testing the motion detection algorithm, we performed preprocessing of the original video sequences to (1) undo the gamma correction effect by transforming the pixel values to be approximately proportional to the light intensity levels and, (2) change brightness levels. First, we scaled the value of each pixel from between 0 and 255 to between 0 and 1 and denoted the scaled video sequence by $u_0$. We then generated an input video sequence as $u(x,y,t) = 10^{u_0(x,y,t)}$ with an approximately scaled version of the light intensity of the original visual scene. The light intensity of the video sequence was furthermore artificially increased by a factor of 10 after every 100 frames, i.e., $u(x,y,t) = 10^{u_0(x,y,t)+k}, k=1,2,3,4$.

To help discern the contents of the visual scenes at every brightness level, Fig. 2(2nd row) shows $log_{10}(u)$ instead of *u* itself, with $log_{10}(1)$ displayed as pure black and $log_{10}(10^6)$ displayed as pure white.

### Constructing video sequences with different contrast levels

We generated the video sequences $u_i(x,y,t) = 100\cdot 10^{c^i(u_0(x,y,t)-0.5)}$, where $c^i$ is the scaling factor for each contrast level. Note that locally, the contrast is different even within a single frame. Here the increase between contrast levels refers to the contrast difference evaluated at the same spatial position for two different time instances.

## Steady-state responses of DNPs

We derive here the steady state response of DNPs in several different configurations, including (1) temporal DNPs with feedforward normalization, (2) temporal DNPs with feedforward and feedback normalization and (3) spatio-temporal DNPs with global feedback. For each configuration, we then illustrate how the DNP parameters affect their steady-state response curve.

### Steady-state I/O of temporal DNPs with feedforward normalization

We first characterize the steady-state response of temporal DNPs with feedforward normalization. The output of the temporal DNP with feedforward normalization is given by

38 $$\begin{aligned} v^i(t) = \frac{{\mathcal {T}}^1 u^i}{{\mathcal {T}}^2 u^i}, \end{aligned}$$

and the steady-state response to a constant intensity value *I* amounts to

39 $$\begin{aligned} v^i(I) = \frac{a_0 + a_1I + a_2 I^2}{c_0 + c_1I + c_2 I^2}. \end{aligned}$$

To ensure that the denominator is always positive, we also assume that the coefficients $a_0, a_1, a_2, c_0, c_1, c_2$ are positive. Therefore,

40 $$\begin{aligned} v^i(0) = \frac{a_0}{c_0}, \end{aligned}$$

determines the response level at the lowest possible light intensity level, and

41 $$\begin{aligned} v^i(\infty ) = \left\{ \begin{array}{ccl} \frac{a_2}{c_2} &{}, &{} \text{ if } c_2 \ne 0, \\ \infty &{}, &{} \text{ if } c_2 = 0, a_2 \ne 0, \\ \frac{a_1}{c_1}&{}, &{} \text{ if } c_2=a_2 = 0, c_1 \ne 0, \\ \infty &{}, &{} \text{ if } c_2 = a_2 = c_1 = 0, a_1 \ne 0, \\ \frac{a_0}{c_0}&{}, &{} \text{ if } c_2 = a_2 = c_1 = a_1 = 0. \end{array} \right. \end{aligned}$$

Clearly, it is not desirable to have $v^i(\infty ) = \infty$. We are interested in the case where $v^i(\infty ) > v^i(0)$, *i.e.*, $\frac{a_2}{c_2} > \frac{a_0}{c_0}$ if $c_2\ne 0$ or $\frac{a_1}{c_1} > \frac{a_0}{c_0}$ if $c_2 = 0$ and $c_1 \ne 0$.

Here we assume that $0 \le a_0 \ll c_0$, and thus $v^i(0) \approx 0$. We also consider normalizing $v^i(\infty )$ to 1. Therefore, either

42 $$\begin{aligned} \frac{a_2}{c_2} = 1, \text{ when } a_2\ne 0 \text{ and } c_2\ne 0, \end{aligned}$$

or

43 $$\begin{aligned} \frac{a_1}{c_1} = 1, \text{ when } c_2=0. \end{aligned}$$

Let $I^{\prime } = {\text {log}}_{10}(I)$, we have

44 $$\begin{aligned} v^i(I^{\prime }) = \frac{a_0 + a_1 10^{I^{\prime }} + a_2 (10^{2I^{\prime }})}{c_0 + c_1 10^{I^{\prime }} + c_2 (10^{2I'})} . \end{aligned}$$

If $$a_2=c_2=0$$, then

45 $$\begin{aligned} \begin{aligned} v^i(I^{\prime })&= \frac{a_0 + a_1 10^{I^{\prime }} }{c_0 + c_1 10^{I^{\prime }} } \\&= \frac{a_0}{c_0 + c_1 10^{I^{\prime }}} + \frac{1}{ \frac{c_0}{a_1}10^{-I^{\prime }} + \frac{c_1}{a_1} }\\&\approx \frac{1}{ \frac{c_0}{a_1}10^{-I'} + \frac{c_1}{a_1} } \end{aligned} \end{aligned}$$

is a sigmoidal function.

Otherwise, we have

46 $$\begin{aligned} \frac{\text {d}v^i(I^{\prime })}{\text {d}I^{\prime }} = \frac{(a_1c_0-a_0c_1)e^{I^{\prime }} + 2(a_2c_0-a_0c_2)e^{2I^{\prime }} + (a_2c_1-a_1c_2)e^{3I'}}{ (c_0 + c_1 e^{I^{\prime }} + c_2 (e^{2I'}))^2 }.\nonumber \\ \end{aligned}$$

Substituting $$a_2 = c_2$$ and $$a_0 \ll c_0$$,

47 $$\begin{aligned} \frac{\text {d}v^i(I^{\prime })}{\text {d}I^{\prime }} = \frac{a_1c_010^{I^{\prime }} + 2c_2 c_010^{2I'} + c_2(c_1-a_1)10^{3I^{\prime }}}{ (c_0 + c_1 10^{I^{\prime }} + c_2 (10^{2I^{\prime }}))^2 }.\nonumber \\ \end{aligned}$$

Figure 15 shows two qualitatively different steady-state response resulted from two sets of parameters. To guarantee $\frac{\text {d}v^i(I^{\prime })}{\text {d}I^{\prime }} >0, \forall I^{\prime }$, *i.e.*, ensuring $v^i(I^{\prime })$ is monotonically increasing, we must have $c_1 \ge a_1$. In Fig. 15A, the “overshoot" in the mid-range light intensity levels is due to $a_1 > c_1$. In contrast, when $a_1 \le c_1$, the steady-state response is sigmoidal.

### Steady-state I/O of temporal DNPs with feedforward and feedback normalization

We now characterize the steady-state response of temporal DNPs with feedforward and feedback normalization. With both feedforward and local-feedback normalization, the output of the DNP can be expressed as

48 $$\begin{aligned} v^i(t) = \frac{{\mathcal {T}}^1 u^i}{{\mathcal {T}}^2 u^i + {\mathcal {T}}^3 v^i}, \end{aligned}$$

and the steady-state response to a constant intensity value *I* is given by

49 $$\begin{aligned} v^i(I) = \frac{ a_0 + a_1I + a_2 I^2}{c_0 + c_1 I + c_2 I^2 + d_1 v^i + d_2 (v^i)^2}. \end{aligned}$$

Therefore, $v^i(I)$ is the real, positive root of the cubic equation

50 $$\begin{aligned}{} & {} d_2 (v^i)^3 + d_1 (v^i)^2 + (c_0+c_1I + c_2I^2)v^i \nonumber \\{} & {} \quad - (a_0+a_1I + a_2 I^2) = 0. \end{aligned}$$

By denoting $A = -(a_0+a_1I + a_2 I^2)$ and $C = (c_0+c_1I + c_2I^2)$, we have

51 $$\begin{aligned} d_2 (v^i)^3 + d_1 (v^i)^2 + Cv^i + A = 0. \end{aligned}$$

If $$d_2 = 0$$, then

52 $$\begin{aligned} v^i = \frac{- C \pm \sqrt{C^2 - 4d_1A}}{2d_1}. \end{aligned}$$

For $v^i$ to be real and non-negative, the coefficients must satisfy $C^2 \ge 4d_1A$, and

53 $$\begin{aligned} v^i = \frac{ \sqrt{C^2 - 4d_1A}-C}{2d_1}. \end{aligned}$$

If $d_2 \ne 0$, then the discriminant

54 $$\begin{aligned} \Delta = \frac{4\Delta _0^3 - \Delta _1^2}{27d^2_2}, \end{aligned}$$

where

55 $$\begin{aligned} \Delta _0&= d_1^2 - 3d_2C, \end{aligned}$$

56 $$\begin{aligned} \Delta _1&= 2d_1^3 - 9d_2d_1C + 27d_2^2A. \end{aligned}$$

In addition, we define

57 $$\begin{aligned} D = \root 3 \of { \frac{\Delta _1 + \sqrt{-\Delta }}{2} }. \end{aligned}$$

If $\Delta > 0$ then the cubic equation has 3 real roots. In this case, since $A<0$ by definition, and there must exists a positive root with value between 0 and 1, we must have the other 2 roots negative. Therefore, the steady-state solution must be the positive root that amounts to

58 $$\begin{aligned} v^i = -\frac{1}{3d_2}\left( d_1 + D \frac{-1+\sqrt{-3}}{2} + \frac{\Delta _0}{D\frac{-1+\sqrt{-3}}{2}}\right) . \end{aligned}$$

If $\Delta \le 0$ then the cubic equation has only one real root, and the steady-state response amounts to

59 $$\begin{aligned} v^i = -\frac{1}{3d_2}\left( d_1 + D + \frac{\Delta _0}{D}\right) . \end{aligned}$$

To sum up, the steady-state response of a temporal DNP with feedforward and feedback normalization is given by

60 $$\begin{aligned} v^i(I) = \left\{ \begin{array}{cc} \frac{-C + \sqrt{C^2 - 4d_1A}}{2 d_1} &{} \text{ if } d_2 = 0, \\ -\frac{1}{3d_2}\left( d_1 + D \frac{-1+\sqrt{3}j}{2} + \frac{\Delta _0}{D\frac{-1+\sqrt{3}j}{2}} \right) &{} \text{ if } d_2 \ne 0, \Delta > 0, \\ -\frac{1}{3d_2}\left( d_1 + D + \frac{\Delta _0}{D}\right) &{} \text{ if } d_2 \ne 0, \Delta \le 0 \end{array} \right. \nonumber \\ \end{aligned}$$

where

61 $$\begin{aligned} A&= -(a_0+a_1I + a_2 I^2), \end{aligned}$$

62 $$\begin{aligned} C&= (c_0+c_1I + c_2I^2), \end{aligned}$$

63 $$\begin{aligned} D&= \root 3 \of { \frac{\Delta _1 + \sqrt{-\Delta }}{2} }, \end{aligned}$$

64 $$\begin{aligned} \Delta _0&= d_1^2 - 3d_2C, \end{aligned}$$

65 $$\begin{aligned} \Delta _1&= 2d_1^3 - 9d_2d_1C + 27d_2^2A. \end{aligned}$$

In Fig. 16, we compare the steady-state responses of the single-channel self-feedback DNP with different $d_1$ and $d_2$ values.

### Steady-state I/O of spatio-temporal DNPs with global feedback

Finally, we characterize the steady-state response of spatio-temporal DNPs with global feedback normalization. The response of a spatio-temporal DNP can be expressed as

66 $$\begin{aligned} v^i(t) = \frac{{\mathcal {T}}^1 u^i}{{\mathcal {T}}^2u^i + {\mathcal {T}}^3v^i + {\mathcal {L}}^4 {\textbf{v}}}, \end{aligned}$$

for $i = 1,2,\ldots ,N$. The steady-state response is given by the system of equations

67 $$\begin{aligned} v^i({\textbf{I}}) = \frac{ a_0 + a_1 I^i + a_2 (I^i)^2 }{ c_0 + c_1 I^i + c_2 (I^i)^2 + d_1 v^i + d_2(v^i)^2 + \sum _{k=1}^{N}g^k_1 v^k + \sum _{k=1}^{N}\sum _{l=1}^{N}g^{kl}_2 v^k v^l}, \end{aligned}$$

for $i = 1,2, \ldots ,N$, where ${\textbf{I}} = \left[ I^1, I^2, \ldots , I^N \right]$ is a vector of constant intensity values that are presented to each photoreceptor *i*, and

68 $$\begin{aligned} g^k_1= & {} \int _{{\mathbb {R}}} h^{k4}(t)dt, k = 1,2, \ldots , N, \end{aligned}$$

69 $$\begin{aligned} g^{kl}_2= & {} \int _{{\mathbb {R}}^2} h^{kl4}(t_1,t_2)dt_1dt_2, k, l = 1, 2,\ldots ,N. \end{aligned}$$

After rearranging terms, the steady-state response of the spatio-temporal DNP should satisfy the following equations

70 $$\begin{aligned} \begin{aligned}&d_2 (v^i)^3 + \sum _{k,l} g^{kl}_2 v^k v^l v^i + d_1(v^i)^2 + \sum _{k=1}^N g^{k}_1v^k v^i \\&\quad + \left( c_0 + c_1 I^i + c_2 (I^i)^2\right) v^i - \left( a_0 + a_1 I^i + a_2(I^i)^2\right) = 0, \end{aligned} \end{aligned}$$

for $i = 1, 2, \ldots , N$. The zeros can be found by using Powell’s hybrid method (Powell 1970) efficiently.
