# Supplementary material for: Divisive normalization processors in the early visual system of the Drosophila brain
Source: Biol Cybern. 2023 Sep 13;117(6):411–31. doi: 10.1007/s00422-023-00972-x (PMC10752861; doi:10.1007/s00422-023-00972-x)
Supplement: Supplementary file 16 — (pdf 477 KB) [file 422_2023_972_MOESM16_ESM.pdf]

Supplementary Information.

Supplementary Videos S1-S3: Comparison of motion detected in a visual sequence shot at 240 fps. S1 corresponds to Figure 9. (1st column) A 240 fps visual sequence. (2nd column) Motion detected by the phase-based motion detector. (3rd column) Motion detected by the motion energy algorithm. (4th column) Motion detected by the MR-flow algorithm. (5th column) Motion detected by the RAFT algorithm. Direction of detected motion is indicated by the common color coding convention (Baker et al, 2011) as well as by arrows on a sparser grid.

Supplementary Videos S4-6: Comparison of motion detected in a "raw" visual sequence shot at 60 fps. S4 corresponds to Figure 10. (1st column) A 60 fps "raw" visual sequence. Pixel values are proportional to light intensity. (2nd column) The video sequence with gamma correction of 2.2. (3rd column) Motion detected by the phase-based motion detector. (4rd column) Motion detected by the motion energy algorithm. (5th column) Motion detected by the MR-flow algorithm. (6th column) Motion detected by the RAFT algorithm. Direction of detected motion is indicated by the common color coding convention (Baker et al, 2011) as well as by arrows on a sparser grid.

Supplementary Videos S7-9: Motion detection by the phase-based motion detector on 240fps video sequences subject to additive white Gaussian noise. (top row) from left to right: 1) Original video sequence, no noise added. 2-6) Video sequences with 30dB, 25dB, 20dB, 15dB and 10dB SNR, respectively. (bottom row) Motion detected by the phase-based motion detector from the video sequences on the top row and same column.

Supplementary Videos S10: Evaluation of motion detection using a cascade of two DNPs. S10 corresponds to Figure 13. (top left) The brightness of the video sequence increases by a factor of 10 after every 100 frames. The resulting video sequence has brightness that spans 5 orders of magnitude. (top right) DNP output without the MVP feedback block. Red arrows indicate the motion detected from the DNP output. (bottom left) DNP output with the MVP feedback block. Red arrows indicate the motion detected from the DNP output. (bottom right) The output of the adaptive feedback DNP. Red arrows indicate the motion detected. The output of this DNP is mostly invariant with respect to the input brightness level.

Supplementary Videos S11-15: Applying a cascade of two DNP processing blocks to detect motion in natural scenes. (top row) 1st column: original video sequence. 2nd column: Output of the DNP without MVP block. 3rd column: Output of the DNP with MVP block. 4th column: Output of the DNP with adaptive feedback block. (bottom row): Motion detected in the original video sequence or the outputs of the DNPs above.
